# The Search for Species Flocks in Marine Benthic *Homoeocladia* spp. (Diatomeae: Bacillariales). I. Variations on Three Themes, Seventeen New Species

**DOI:** 10.3390/plants12234073

**Published:** 2023-12-04

**Authors:** Christopher S. Lobban, Britney Sison, Matt P. Ashworth

**Affiliations:** 1 Division of Natural Sciences, University of Guam, Mangilao, GU 96923, USA; 2 SEAS Alliance Program, University of Guam, Mangilao, GU 96923, USA; 3 UTEX Culture Collection of Algae and Department of Molecular Biosciences, University of Texas at Austin, Austin, TX 78712, USA; mashworth@utexas.edu

**Keywords:** biodiversity, conopeate *Nitzschia*, coral reefs, morphology, species flocks, ultrastructure

## Abstract

Exploration of the diversity in the diatom genus *Homoeocladia* across Micronesia revealed several clusters of undescribed species based on variations around several characters. Using ultrastructural data from scanning electron microscopy, we describe seventeen new species in three of these morphological groups. (1) A group with external thickenings includes eight new species with costae and/or bordered areolae on valve face and/or conopea and/or peri-raphe zone, and one with similar areolae but no ornamentation; this group includes the previously described *H. jordanii*. (2) Large, linear species, resembling *H. asteropeae* and *H. tarangensis*; we describe three new species close to the latter. (3) A sinuous-areolae group includes five new species with areola openings shaped like “~”, “s”, or “z” on the valve and/or girdle bands, or both, and leads to reconsideration of the diagnosis of *Homoeocladia schefterae* and the recognition that the globally widespread species in this complex is *H. coacervata* sp. nov. The three groups are based solely on morphology and no genetic relationships are implied within or between the groups, other than having the characteristics of the recently redefined genus *Homoeocladia*. However, the high diversity of species in *Homoeocladia* suggests the genus is a good candidate to test for species flocks in this region and in at least one other comparable location, incorporating DNA sampling through either culturing or metabarcoding.

## 1. Introduction

Diatoms are unicellular photosynthetic eukaryotes (algae) defined by a silica shell and yellow-brown auxiliary pigments [1,2,3,4]. Their abundance contributes a substantial fraction of the oxygen in the atmosphere and a large fraction of primary production in aquatic habitats [5], passing carbon up the food chain and directly fueling migrations of shorebirds that eat biofilm [6]. Their fossil deposits—diatomite—are mined for products ranging from insecticides to dynamite and used for stratigraphic analyses. Modern forms are used as environmental monitors, especially for water quality analysis [7,8], and freshwater diatom floras are thus much better known than marine floras [9], particularly benthic tropical floras of coral reefs and mangroves [10]. The discovery of tropical benthic diatom diversity has been impeded not only by a lack of financial drivers but also by a pragmatic tendency to fit tropical specimens into European taxa.

A species flock is an accumulation of a relatively large number of closely related species confined to a narrowly circumscribed area. Besides bird species such as Darwin’s finches in the Galápagos Islands, the most famous examples are cichlid fishes and crustaceans in East African Rift Lakes and other ancient lakes [11,12,13]. It is an evolutionary hypothesis involving radiations of species, defined by three criteria: (a) high diversity of species that are (b) closely related (monophyletic), and (c) geographically constrained (high endemicity) [14,15]; radiations may be adaptive or nonadaptive [13]. To these criteria for ‘core flocks,’ Eastman and McCune [16] and Lecointre et al. [17] added (d) morphological/ecological diversity and (e) habitat dominance to define ‘full flocks’ [13]. Since microbes can only be observed in tiny samples, it becomes impossible to establish endemicity because there is always an overwhelming absence of evidence [18]. Nevertheless, endemicity in general and species flocks in particular have been anticipated for eukaryotic microorganisms, including freshwater diatoms [19] and direct evidence for a diatom species flock was presented by Stelbrink et al. [18] by combining molecular, fossil, and biogeographical data on *Aneumastus* spp. from the ancient Lake Ohrid.

The broader question of endemicity among microbes has a much longer history of debate. The ubiquity (EiE) “hypothesis,” originally proposed for bacteria [20], held that, ‘Everything is everywhere, but the environment selects’ [21]. Finlay and coworkers [22,23,24] extended the hypothesis to include all cells less than 1 mm diameter, i.e., almost all eukaryotic microbes. O’Malley [25] and Williams [26] explained why EiE does not meet the criteria of a hypothesis. EiE has several corollaries, among them that endemicity will be very low because any new species should be rapidly dispersed and turn up in all suitable habitats, thus minimizing geographic differences between similar habitats. Findlay et al. [22] (p. 261), speaking about freshwater diatoms, categorically dismissed endemicity: “The argument in favour of endemic diatom species is untenable, because it is not possible to disprove their existence elsewhere in the biosphere.” Of course, this argument also works in reverse, i.e., that it is not possible to prove that everything is everywhere (that the environment would allow growth). Even Fenchel and Finlay [27] (p. 1969) allowed that “some examples of real biogeography of limnic or terrestrial protists may turn out to be correct.” Our search for species flocks presumes that *Homoeocladia* has biogeography. Williams [26] suggested presupposing that everything is endemic; in our view, this is a wise strategy because, even if it is wrong, we will find more species than if we presume everything is everywhere. In Mann and Vanormelingan’s [28] (p. 418) intermediate dispersal hypothesis, “long-distance dispersal seems in many cases to be effective enough for species to establish across large geographic areas, [but] gene flow is not frequent enough over the short term to prevent populations from diverging.” Sabbe et al. [29], Vyverman et al. [30] and Vanormelingen et al. [31] made the case for diatom endemicity, especially in Australasian freshwater taxa; later Williams and Kociolek [32] argued that the distribution of taxa above the species level could be evidence of endemicity (they used an Order, again for austral freshwater taxa, but it had also been shown with genera, e.g., *Actinella* [29]).

In contrast to freshwater lakes, marine benthic diatom floras are not separated into clearly bounded water bodies and have been expected to show less endemicity, even though (as noted by Williams and Kociolek [32]) seaweed floras clearly display endemicity. Recently, however, Lobban and Santos [33] used Williams and Kociolek’s [32] approach to infer regional endemicity in western Pacific/eastern Australia *Licmophora* floras compared to Atlantic/Mediterranean floras in the literature and Riaux-Gobin et al. [34] compared small Achnanthales floras between West Atlantic and Indo-Pacific Ocean basins and identified ‘potential endemics’ among different regions of the Indo-Pacific.

Our ultrastructural studies of conopeate *Nitzschia* spp. [35], now separated as *Homoeocladia* [36], revealed high species diversity in a small number of samples from Guam, Mariana Islands, and a different set of species were glimpsed in Yap, Federated States of Micronesia [37]. These results suggested that there might be enough diversity to look for species flocks within the region. Micronesia extends 4000 km across the western Pacific Ocean from Palau to the Marshall Islands (Figure 1). Comparisons with other regions will not be possible until similar ultrastructural studies can be conducted elsewhere. We collected additional samples from Guam to provide a broader baseline and examined existing herbarium accessions and new samples from Palau to the Marshall Islands.

## 2. Materials and Methods

Materials were drawn from the Diatom Collection of the University of Guam Herbarium (GUAM), prepared and examined following standard protocols [38]. In short, samples had been collected by hand from the shore or via scuba diving, preserved in formalin or alcohol. Aliquots were rinsed of preservative and boiled in 50:50 sample + water: concentrated nitric acid for 10 min, settled for 24 h and rinsed 10× with distilled water. If calcareous material was present, a pretreatment with hydrochloric acid was given in a large flask. The remaining residue, largely free of organic matter, was subsampled onto coverslips and filter paper. The former were made into permanent mounts on glass slides with Naphrax for light microscopy (LM) [Olympus *i*80 microscope with differential interference contrast (DIC) optics] and the latter cut out, stuck to aluminum stubs and coated with gold for examination by scanning electron microscopy (SEM) [Phenom XL G2 desktop SEM with optional eucentric (tilting) stage and/or a Zeiss SUPRA 40VP SEM]. LM was of little use because most taxonomic characters can be seen only in SEM.

**Figure 2:**
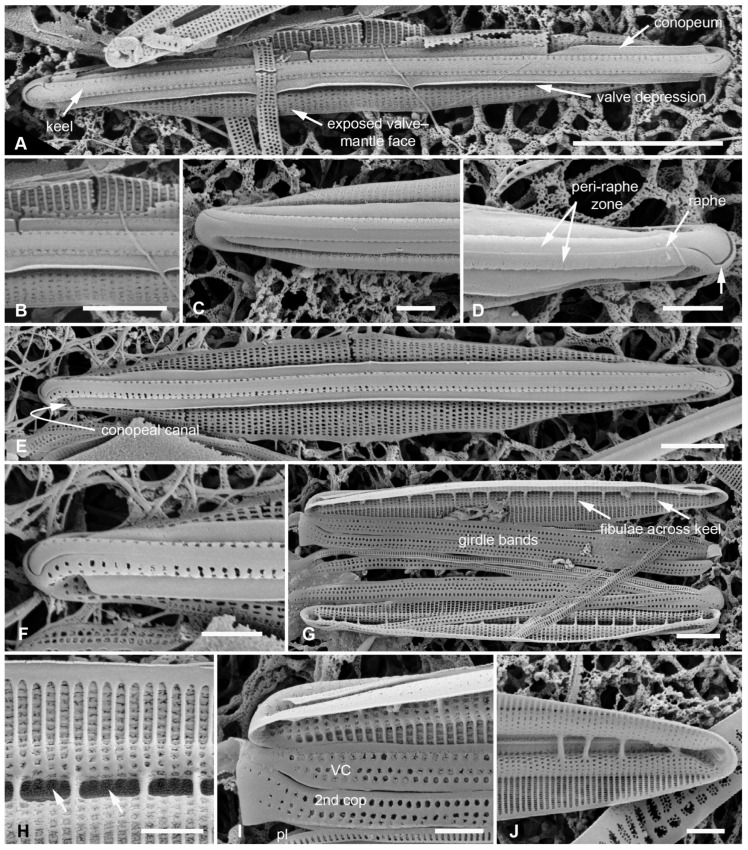
(**A**–**I**). *Homoeocladia marshallensis* sp. nov. in SEM (sample M2-13), also showing structure of the *Homoeocladia* frustule; (**J**) *H. taygeteae*. (**A**,**B**) Holotype specimen, showing external and part of internal valve morphology. (**C**) External detail of apex, showing areolae in exposed valve face and peri-raphe zone. (**D**) External apex of a more rostrate specimen, arrow points to hook in raphe terminal fissure. (**E**,**F**) Valve with eroded areolae, external valve, and detail of apex. (**G**) Frustule showing internal valve surfaces and girdle bands. (**H**) Detail of valve interior showing costae under the exposed valve face and absence of longitudinal break in striae. Areolae can be seen in the peri-raphe zone (arrows). (**I**) Detail of (8), showing girdle bands: valvocopula (VC), 2nd copula, and pleura (pl). (**J**) *H. taygeteae* for comparison of internal costae; the areolae in this species are apically oriented slits. Scale bars: (**A**) = 5 µm, (**B**,**E**,**G**,**H**) = 2 µm, (**C**,**D**,**F**,**I**,**J**) = 1 µm.

Lobban and Ashworth [36] redefined *Homoeocladia*, originally erected by Agardh [39] for the tube-dwelling species *H. martiana* C.Agardh, changing the criteria to accommodate the valve structure rather than mucilage secretions, to pull out the bilaterally symmetrical subset of conopeate *Nitzschia* spp. These are characterized by a central keel bordered by conopea over valve depressions ([35], Figure 6), contrasting the asymmetrical species that can have Hantzschioid cell division ([40], Figure 2). The structure of the *Homoeocladia* frustule is labeled in Figure 2A–I; a cross-section diagram was published in ([35], Figure 6). The silica shells of diatoms comprise two valves connected by several girdle bands. The valves have an abrupt or gradual transition from the valve face to the mantle; the valve face is often flat or uniformly curved (resembling the shape of an overturned boat hull), and in Nitzschioid diatoms there is a keel, usually not along the midline. In *Homoeocladia*, the keel is along the midline and the valve face is depressed alongside each side of the keel and covered by long flaps of silica, the conopea (Figure 2A). A conopeum arises at or near the top of the keel, forming a covered conopeal canal, open at the ends and along the sides (Figure 2A,E). The keel sides are connected internally across the bottom by fibulae (Figure 2G); the fibulae and valve shape are visible in light microscopy (see Figure 12D, below). The top surface of the keel, called the peri-raphe zone, is part of the valve surface and often shows areolae (Figure 2D). The keel is sometimes higher near the apices forming a keel crest (see Figure 12A–H, below). A single raphe branch extends from apex to apex; at the apices it is deflected to the ontogenetically primary side before its terminal fissure hooks toward the secondary side (Figure 2D arrow, Figure 16B). The girdle bands comprise one or more similar copulae and sometimes some distinctly different pleurae; in *Homoeoeocladia,* we found the 1st copula (=valvocopula, adjacent to the valve) and 2nd copula were broadly similar and in many cases we were able to distinguish at least one narrow pleura (Figure 2G,I).

Since the work reported in [35], we have been able to study raw materials from new habitats (especially mangroves and biofilms) locally and from other islands across Micronesia (Figure 1, Table 1), including Republic of Palau; Saipan (Commonwealth of the Northern Mariana Is.); Yap, Chuuk and Pohnpei (Federated States of Micronesia); and Majuro and Bikar Atolls (Republic of the Marshall Is.). A new desktop SEM in 2022 allowed us to resume work in Guam on this genus, which had previously been limited to study with a full-sized SEM at U. Texas at Austin. Nevertheless, time on the latter revealed additional fine details and we encountered more rare species.

**Figure 3:**
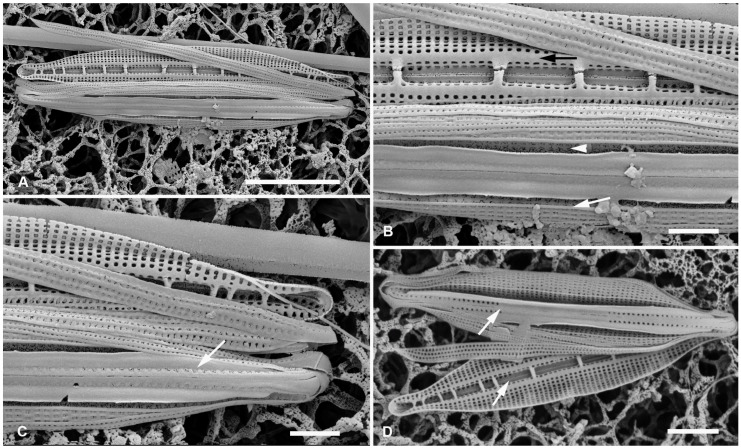
*Homoeocladia majurana*, sp. nov. in SEM (sample M2-13). (**A**–**C**) Holotype. (**A**) Entire frustule. (**B**) Detail of central portion showing infilling of areolae in the middle of the peri-raphe zone (compare (**B**) and (**D**)); costa along boundary of valve depression (white arrow); weak costae on exposed valve face and girdle bands, regular infilling of internal side of exposed valve face, and internal longitudinal break in striae at base of keel (black arrow). Areolae in valve depression visible in (**B**) (arrowhead). (**C**) Detail of apex, note areolae in peri-raphe zone (arrow). (**D**) Somewhat-eroded specimen showing internal and external valve faces and girdle bands; here, the infilling of areolae in the middle of the peri-raphe zone is clear in both external and internal views (arrows point to transition). Scale bars: (**A**) = 5 µm, (**D**) = 2 µm, (**B**,**C**) = 1 µm.

Terminology follows Lobban et al. [35] and references therein, but we need to clarify what we will call the external thickenings present on one group of species. In our earlier paper, we used the informal term *ribs* for the longitudinal lines we saw on a few species. *Costa* is a broad term without a specific link to ultrastructure or morphogenesis. For some *Homoeocladia*, we use *bordered areolae* to describe a specific ultrastructural morphology of unknown morphogenesis, and we use *costae* only for sharply defined, continuous transverse (Figure 2H) or longitudinal thickenings (Figure 5). In the character states for the boundary along the edge of the valve depression, the distinction between a raised costa or a flat break was not always clear and we defined only two character states, boundary present or absent (see Discussion). Some species displayed low ridges on the valve surface, not sufficiently thick or sharply defined to be costae; these we simply describe as low ridges (compare in *H. majurana* sp. nov. the costa on the boundary of the valve depression with the low ridges on the valve face and copulae, Figure 3B). Costae on conopea are a significant character, clearly seen in *H. corrugata* sp. nov. and *H. equitorquis* sp. nov., but we do not include in this definition the single apparent thickening on the outer edge of a conopeum because it may just be a curling artefact (e.g., *H. celaenopsis* sp. nov.).

For comparative purposes, we grouped the 17 species into three visual themes that do not necessarily represent a single character nor imply any homology.

## 3. Results

### 3.1. Theme 1: “Bordered Areolae”

Although we use “bordered areolae” as a shorthand for this theme, the range of characters includes external thickenings longitudinally and/or transversally separating areolae on valve or girdle bands, and longitudinal costae on the peri-raphe zone, conopea, and valve and/or girdle bands.

A representative species already described [35] is *Homoeocladia jordanii* (Lobban, Ashworth, Calaor and E.C.Theriot) Lobban and Ashworth, bearing longitudinal costae on the valve surfaces and girdle bands (see Figure 5). We also described *H. alcyoneae* (Lobban, Ashworth, Calaor and E.C.Theriot) Lobban and Ashworth, which had similar areolae but no external thickenings, except for one specimen ([35], Figure 82), which a re-examination showed to be a different species (described below as *H. contraria* sp. nov.). Characters common in the group (also shared with others and some exceptions): valves lanceolate and rostrate, small (mostly 15–30 µm long; one exception, *H. radiata* sp. nov.), striae of relatively large, occluded areolae ([35], Figure 13; character state = 3) and exposed as a single line of areolae in the peri-raphe zone (one exception, occluded in *H. ngiwalensis* sp. nov.) (Table 2).

We begin with a new undecorated species resembling *H. alcyoneae* and have arranged the rest roughly according to the extent of various patterns of external thickenings from the simplest to most complex, without any implication of lineage either from one to another or of the group as a whole.

#### 3.1.1. *Homoeocladia marshallensis* Lobban, Sison and Ashworth, sp. nov.

Figure 2A–I

Diagnosis: Areolae on valve face and in peri-raphe zone occluded externally, external thickenings absent; differing from *H. alcyoneae* internally by the infilling pattern of the basal lamina.

Holotype: Figure 2A,B, from specimen on stub 1673, according to Article 40.5 of the International Code of Nomenclature for algae, fungi, and plants (Shenzhen Code) [41].

Registration: http://phycobank.org/104090.

Type locality: MARSHALL ISLANDS: Majuro Atoll, Laura, lagoonside beach, 07°09.530′ N, 171°02.336′ E; cyanobacterial tuft with coralline red alga *Amphiroa*, within 1m below low water, sample M2-13. Coll. ca. 30 May 2022, Voneric Boktok, College of the Marshall Islands.

Morphology: Valves lanceolate, weakly rostrate, 20–24 µm long, 2.8–3.1 µm wide, striae 49–50 in 10 µm, parallel, quadrate areolae closed externally by hymenes (Figure 2B–D,H); fibulae 9 in 10 µm (Figure 2G). Keel raised above conopea without elevation of the raphe slit; 1–2 rows of areolae in the peri-raphe zone, continuing to apex (Figure 2B–F,I). Internal silicification of basal lamina of the *taygeteae* type, infilled in the valve depression to completely surround the areolae, under exposed valve/mantle face thickened only along virgae (Figure 2H,I). Figure 2J shows internal face of *H. taygeteae* (Lobban, Ashworth, Calaor and E.C.Theriot) Lobban and Ashworth from same locality. No break in areolae at base of keel (Figure 2H). Girdle bands: two copulae with three rows of large areolae, wider space between advalvar row and others (Figure 2G,I); broad ligule on 2nd copula fits gradual taper of open end of valvocopula; pleura with single line of elongated slits (Figure 2G,I).

Etymology: Named for the archipelago where it was found.

Comments: Externally, *H. marshallensis* is very similar to *H. alcyoneae* in the large pores and absence of external thickening but it is clearly distinguished by the pattern of infilling; there are small differences in the metrics, particularly the length (Table 2). It occurred among abundant *Homoeocladia coacervata* sp. nov. and *H. dagmannii* (Lobban, Ashworth, Calaor and E.C.Theriot) Lobban and Ashworth (Appendix A).

#### 3.1.2. *Homoeocladia majurana* Lobban, Sison and Ashworth, sp. nov.

Diagnosis: Distinguished from *H. marshallensis* by costa along edge of valve depression and internal silicification uniformly infilled except for longitudinal break at base of keel.

Holotype: Figure 3A–C, from specimen on stub 1673, according to Article 40.5 of the International Code of Nomenclature for algae, fungi, and plants (Shenzhen Code) [41].

Registration: http://phycobank.org/104091.

Type locality: MARSHALL ISLANDS: Majuro Atoll, Laura, lagoonside beach, 07°09.530′ N, 171°02.336′ E; cyanobacterial tuft with coralline red alga *Amphiroa*, within 1m below low water, sample M2-13. Coll. ca. 30 May 2022, Voneric Boktok, College of the Marshall Islands.

Morphology: Valves linear-lanceolate, 19–22 µm long, 3.0–3.5 µm wide, apices rostrate; striae parallel, 54 in 10 µm (Figure 3A,D), areolae quadrate, occluded by hymenes, on exposed valve–mantle face bordered by low ridges (Figure 3B,C). Fibulae 8 in 10 µm, irregularly spaced (Figure 3A,D). Longitudinal costa along edge of valve depression (Figure 3C), no other costae or ridges on valve face. Large areolae in the peri-raphe zone, but apparently occluded or infilled completely near the middle (Figure 3B,D). Internally, uniform infilling around areolae except for longitudinal break in striae at base of keel (Figure 3B,C). Girdle bands with 2–3 rows of large pores separated by low ridges (Figure 3C).

Etymology: Epithet derived from Majuro Atoll, where it was collected.

Comments: This species was present in the same sample as *H. marshallensis*, both uncommon, among abundant *H. dagmannii* and *H. coacervata* sp. nov.

#### 3.1.3. *Homoeocladia radiata* Lobban, Sison, and Ashworth, sp. nov.

Figure 4A–E

Diagnosis: Rostrate valve with strongly radiate striae near apex; curving, longitudinal low ridges along the vimines on mantle and copulae.

**Figure 4:**
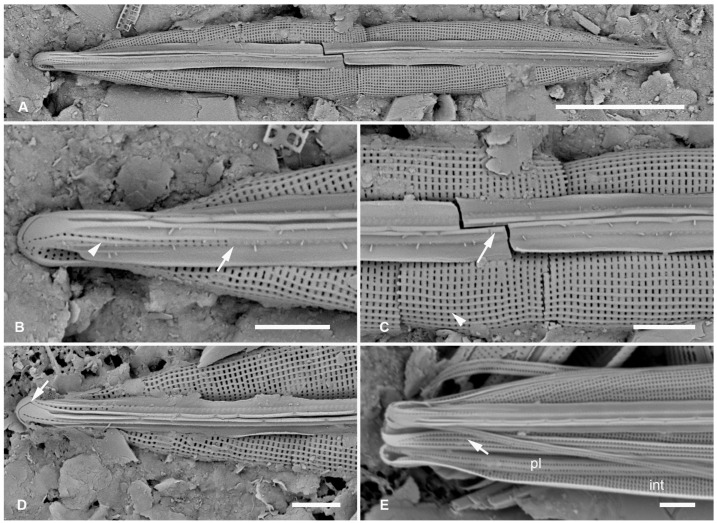
*Homoeocladia radiata*, sp. nov. in SEM (sample Y26C). (**A**–**C**) Holotype, valve in external view. (**A**) Entire specimen. (**B**) Detail of rostrate apex (slightly oblique) with strongly radiate striae and areolae in peri-raphe zone infilled before the keel crest (arrow), also showing short costa and single line of pores on apex (arrowhead). (**C**) Central portion showing costae on vimines (arrowhead) and flanking raphe (arrow). (**D**) Vertical view of valve apex showing hooked terminal raphe fissure. (**E**) Low resolution image of portion of frustule showing regular interior (int) infilling of valve striae, pleura (pl), and costae on copula (arrow). Scale bars: (**A**) = 10 µm, (**B**–**E**) = 2 µm.

Holotype: Figure 4A–C, from specimen on stub 1345, according to Article 40.5 of the International Code of Nomenclature for algae, fungi, and plants (Shenzhen Code) [41].

Registration: http://phycobank.org/104092.

Type locality: FEDERATED STATES OF MICRONESIA: Yap, Tarang (“O’Keefe’s Island”), 09°31.502′ N, 138°07.944′ E; subtidal sediments from 15 m depth, sample Y26C. Coll. 25 Sep. 1988, C.S. Lobban and M. Schefter.

Morphology: Valve lanceolate with long rostrum and shallow keel crest, length 39–51 µm, width 6 µm, striae parallel in middle becoming strongly radiate toward apices (to 15° from perpendicular), 50 in 10 µm (Figure 4A–D); fibulae not seen. Raphe between prominent costae that extended over the keel crest. Two short costae near apex, separating edge of keel from top row of areolae that become a row of small pores going down to the mantle, the other costa partially between the two rows of areolae (Figure 4B,E). The paired areolae extended a short distance toward the center from the keel crest but the rest were filled in. Areolae on exposed valve–mantle face opening externally by transapical slits, on the mantle separated by low ridges on the vimines. Internal view only glimpsed in Figure 4E, internal silicification appears regular. Girdle bands probably comprising two wider copulae with three rows of areolae separated by low ridges (Figure 4E, arrow) and a pleura with 1 row of areolae and no ridges (Figure 4E).

Etymology: Named for the strongly radiating striae, the prominent feature.

Comment: Areolae in this species are unlike those of other species in this group and more like those of *H. dagmannii*, which also has some low ridges on the mantle.

#### 3.1.4. *Homoeocladia jordanii* (Lobban, Ashworth, Calaor and E.C.Theriot) Lobban and Ashworth

Figure 5

**Figure 5:**
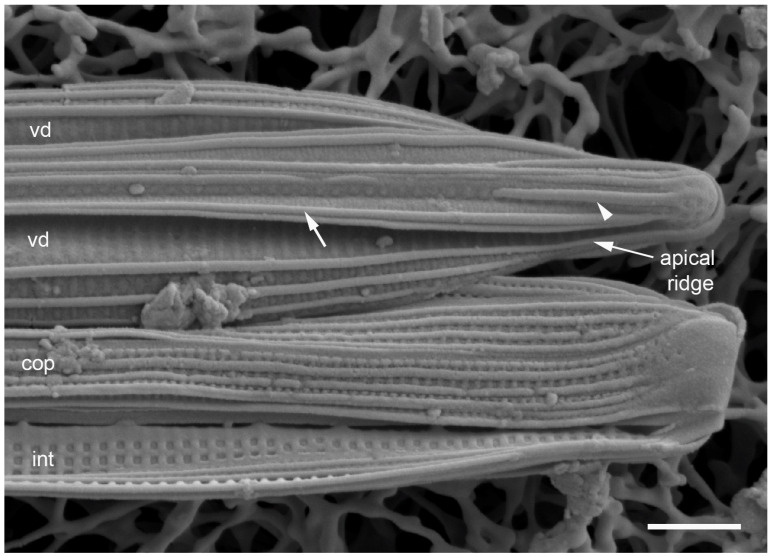
*Homoeocladia jordanii* in SEM (sample GU52G-A). One pole of frustule showing external valve and girdle bands (cop) with costae. Costae occur on exposed valve face, on keel at apex (arrowhead), and on edges of conopea (arrow). Most medial costa, continuation of apical ridge on valve border, is the boundary of the valve depression (vd). Internal surface (int) shows longitudinal break in striae at base of keel. Scale bar = 1 µm.

This species, described from Guam samples ([35], p. 213, Figures 51–55), is distinguished by two thick longitudinal costae on valve face, not between every line of areolae, costae on apex, conopea and girdle bands; transverse thickenings absent. Areolae occluded on the outer side. Internal infilling regular, with longitudinal break at base of keel (claimed but not clearly shown in 2019). An unpublished image (Figure 5) is provided for direct comparison with the next species and it shows the longitudinal break. We now interpret the most medial costa as being the edge of the valve depression, so that, in contrast to *H. ngiwalensis* sp. nov. (below), the valve depression does not follow the indentation of the conopeum (see Section 4.1 Discussion: Morphological characters).

#### 3.1.5. *Homoeocladia ngiwalensis* Lobban, Sison and Ashworth, sp. nov.

Figure 6A–F

Diagnosis: Hymenate areolae on valve face but occluded in peri-raphe zone. Conopea and valve depression narrowed in mid-section; longitudinal costae between each row of areolae on the exposed valve–mantle face.

Holotype: Figure 6A,B, from specimen on stub 1573, according to Article 40.5 of the International Code of Nomenclature for algae, fungi, and plants (Shenzhen Code) [41].

Registration: http://phycobank.org/104093.

Type locality: PALAU: Babeldaob Island, Ngiwal State, Lekes mangrove, 07°31.867′ N, 134°37.183′ E, in mud scraped from red mangrove pneumatophores, sample PW2021-4-7. Coll. 7 July 2021, Kebang Ngeraklang, Palau Community College.

Morphology: Valves elliptical-lanceolate, 24 µm long, 4–5 µm wide, quadrate areolae occluded at outer surface in parallel to radiate transverse striae 52 in 10 µm (Figure 6A–D,F). Hymenate areolae on valve face and peri-raphe zone with no row of small pores on apex (Figure 6B,D). Frustules are strikingly decorated with longitudinal, possibly cylindrical costae, which occur on keel, conopeum, exposed valve face, and girdle bands (Figure 6B–D). Several short costae at apex of keel and on wide part of conopea; raphe in a thickened ridge and bordered by a pair of costae (Figure 6C,D). Costae on exposed valve face form a series of curved lines along the vimines, even when the striae appear parallel (Figure 6A–D). Conopea and valve depressions narrowed in the middle (Figure 6D–F), apical ends of conopea tapering into keel as in *H. jordanii* (no “nose”—contrast Figure 6B with *H. ngesaolensis* Figure 12G below). Fibulae, average 10 in 10 µm, closer along the center part (16 in 10 µm), where conopea are narrow (Figure 6E,F). Internally, *taygeteae* pattern of infilling (Figure 6E,F), only vimenes thickened under exposed valve/mantle, basal layer silicified all around areolae under the depression, ranging from 4 to 5 areolae in the widest part of the conopeum to 1 under the narrowest part (Figure 6F). Infilled break in the areolae along the base of the keel (Figure 6E,F). Girdle bands: two copulae with 3–4 rows of pores and 4–5 longitudinal costae between and outside the rows (Figure 6A,B,F), narrower pleura with two rows of pores, three costae.

Etymology: Named for the type locality, Ngiwal, Babeldaob Island, Palau.

Other records: GUAM: GU58G-4A! (Achang mangroves, intertidal mud scraped from mangrove root at reef edge)

Comments: *H. jordanii* (Figure 5) is slightly smaller with a costa in middle of each conopeum and a regular pattern of internal infilling (Table 2).

#### 3.1.6. *Homoeocladia contraria* Lobban, Sison and Ashworth, sp. nov.

Figure 7A–F

Synonym: *Nitzschia alcyoneae* Lobban, Ashworth, Calaor and E.C.Theriot [35] (in part)

**Figure 7:**
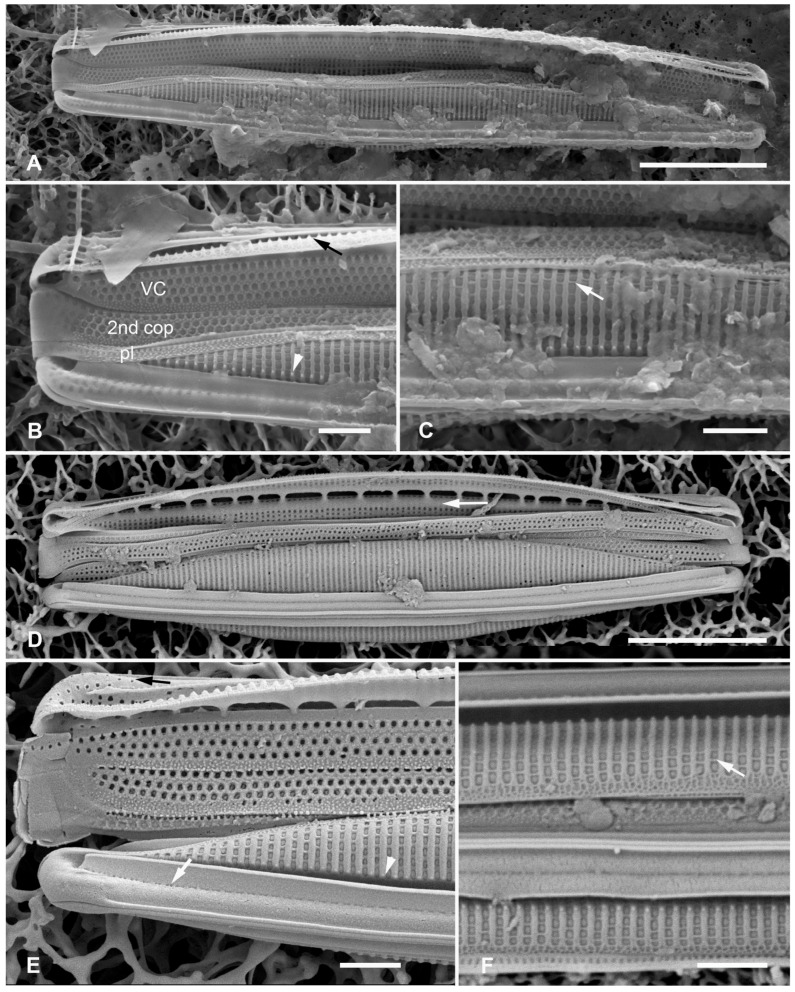
*Homoeocladia contraria* sp. nov. in SEM. (**A**–**C**) Holotype from Guam (GU52K-7). (**A**) Entire frustule. (**B**) Detail of apex, showing costae on exposed valve face with papilla at edge of valve depression (arrowhead), elevation indicated in edge view (black arrow); girdle bands [valvocopula (VC), 2nd copula, pleura (pl)] with bordered areolae; reproduced from *Phytotaxa* [35] with permission of the copyright holder, Magnolia Press. (**C**) Central portion of frustule showing some thin borders on vimines toward the distal end of striae (arrow). (**D**–**F**) Specimens from Palau. (**D**) Entire frustule showing interior and exterior valve faces and girdle bands, longitudinal break in striae (arrow) (PW2021-4-7). (**E**) Apex (PW2009-46 specimen), showing small openings of areolae in peri-raphe zone (white arrow), second line of areolae on apex of the more eroded valve (black arrow) and papillae on transverse costae (arrowhead). (**F**) Detail of mid portion showing thin borders on vimines toward the distal end of striae (arrow) and pitted border (GU68D-1B). Scale bars: (**A**,**D**) = 5 µm, (**B**,**C**,**E**,**F**) = 1 µm.

Diagnosis: Areolae in peri-raphe zone almost infilled, transverse costae on valve–mantle face, the more distal areolae with more or less distinctly thickened vimines, girdle bands with bordered areolae.

Holotype: Figure 7A–C from stub 230, according to Article 40.5 of the International Code of Nomenclature for algae, fungi, and plants (Shenzhen Code) [41].

Registration: http://phycobank.org/104094.

Type locality: U.S.A. Guam: Outhouse Beach, Apra Harbor, 13°27.840′ N, 144°39.360′ E, farmer fish turf [*Stegastes nigricans* (Lacépède, 1802)], ca. 5 m deep, sample GU52K-7. Coll. 3 May 2009, C.S. Lobban and M. Schefter.

Morphology: Valves lanceolate, weakly rostrate, 24–31 µm long, 5–6 µm wide, striae 50 in 10 µm (Figure 7A,D), fibulae 9–10 in 10 µm (Figure 7D), irregularly spaced. Striae of quadrate to transapically elongated areolae occluded at the outer surface, virgae thickened into transapical costae each with a papilla on the proximal end (Figure 7B,C,E,F); shallower thickenings sometimes on distal vimines (Figure 7B,F); pitted border on valve (Figure 7F). Areolae in peri-raphe zone visible only as slits at edge of conopea, continuing to the apex (Figure 7B,E), along with second row of areolae (clearly visible only in a partially eroded valve, Figure 7E). Raphe raised between two costae (Figure 7E,F). Conopea uniformly wide, smooth. Internally, basal silica layer infilled uniformly, leaving small round foramina; longitudinal break in striae at base of keel (Figure 7D,E). Girdle bands: broad valvocopula and 2nd copula with 3–4 rows of large pores decussately arranged and with papillate borders, pleura narrow (Figure 7B,E).

Etymology: Latin *contrarius*, against, opposite, with reference to our earlier misidentification.

Additional records: GUAM: GU68-1B!; PALAU: Babeldaob Island, Ngaremlengui State, dock at Bkulangriil, PW2009-46! sand sample rich in *Carinasigma*; Lekes mangrove, PW2021-4-7!, mud from red mangrove pneumatophores.

Comments: The Guam specimen was misidentified as *Nitzschia alcyoneae* in ([35], Figure 82). There was some variation in the thickening of the vimines, sometimes not clear in the desktop SEM images, but we could not find additional characters to separate those from *H. contraria*. The presence of areolae on the keel at the apex also seemed variable but can be explained by occluded areolae being indistinguishable from the surrounding surface, as noted in other species. While faint areolae were evident in the Guam specimen (Figure 7B) and usually not in the Palau specimens, the frustule in Figure 7E shows two valves, the upper, apparently more eroded, displaying the areolae, not evident in the lower, presumably younger valve. However, there does not appear to be a row of small pores.

#### 3.1.7. *Homoeocladia equitorquis* Lobban, Sison and Ashworth, sp. nov.

Figure 8A–I

Diagnosis: Differing from congeners in the combination of large, bordered areolae in the peri-raphe zone, without costae on the valve face, areolae occluded at inner opening.

Holotype: Figure 8A–F, from specimen on stub 1573, according to Article 40.5 of the International Code of Nomenclature for algae, fungi, and plants (Shenzhen Code) [41].

Registration: http://phycobank.org/104095.

Type locality: PALAU, Babeldaob Island, Ngiwal State, Lekes mangrove, 07°31.867′ N, 134°37.183′ E, in mud scraped from red mangrove pneumatophores, sample PW2021-4-7. Coll. 7 July 2021, Kebang Ngeraklang, Palau Community College.

Morphology: Valves lanceolate, rostrate, ca. 17–24 µm long, 3.6 µm wide; striae 50–52 in 10 µm; fibulae 10 in 10 µm, irregularly spaced (Figure 8A). Large areolae in peri-raphe zone individually bordered by U-shaped thickenings open at distal side (Figure 8C). Valve–mantle surface with smaller, transapically elongate areolae with hymenes on the inner side (Figure 8D,E). These areolae interpreted as not bordered, since the outer surface of virgae and vimenes is smooth. The holotype specimen shows one or two longitudinal costae on each conopeum, giving a corrugated appearance (Figure 8C); specimens from Jellyfish Lake, Palau (a marine lake with only small connections to lagoon waters) were smooth (Figure 8H,I). Raphe raised between two ribs (Figure 8G). No boundary along edges of valve depressions except for a short ridge in the rostrum (Figure 8B,H) arrows), nor any external costae; small pores absent from apex; keel crest absent. Internally, regular pattern of infilling, thus inner surface smooth; longitudinal break along base of keel (Figure 8F). Girdle bands also with hymenes on inner side (Figure 8F): two copulae, one with up to six longitudinal rows of areolae in decussate pattern, the other with two rows of elongated areolae; pleura possibly very narrow (Figure 8G), but we could not accurately identify the bands seen in Figure 8A,G.

Etymology: L. *equus* (horse) + *torquis* (collar), compound noun (f.) in apposition, for the shape of the borders around the areolae in the peri-raphe zone.

Additional records: PW2009-22 stub 1520! and PW2009-23 stub 1521!, both Jellyfish Lake, epiphytes on seaweeds associated with red mangrove prop roots (not muddy).

#### 3.1.8. *Homoeocladia corrugata* Lobban, Sison and Ashworth, sp. nov.

Figure 9A–F

Diagnosis: Distinguished by combination of longitudinal costae covering conopea and peri-raphe zone and complex pattern of thickened vimines and marginal pits on exposed valve face.

Holotype: Figure 9A–C, from specimen on stub 1564, according to Article 40.5 of the International Code of Nomenclature for algae, fungi, and plants (Shenzhen Code) [41].

Registration: http://phycobank.org/104096.

Type locality: FEDERATED STATES OF MICRONESIA: Pohnpei, Kitti Municipality, Pehleng, Dauen Nahnsakar mangrove, 6°52.767′ N, 158°9.390′ E, in mud scraped from *Avicennia* pneumatophores, sample PN2-9. Coll. 17 June 2021, Marlin Lee Ling and Brian Lynch, College of Micronesia.

Morphology: Valves 18–20 µm long, ca. 4 µm wide, striae parallel, 52 in 10 µm, fibulae 10 in 10 µm (Figure 9A–C). Raphe bordered by costae (Figure 9B,D). Areolae in the peri-raphe zone possibly infilled, or with hymenes at outer surface (Figure 9C,D). In contrast, hymenes of exposed valve appeared to be close to, but not flush with, inner surface (Figure 9B,E), except for the few unbordered areolae clearly present on apex (Figure 9B,D). Borders around areolae on exposed valve–mantle exterior complicated (Figure 9E, arrows a–d) but, interpreting the hymenes to be near the inner side, more proximal vimines were not thickened but there were distinctly thickened, short longitudinal costae (c) on the distal vimines, those toward the apex continuous with a short apical costa (Figure 9B,D arrows). Virgae seemed to be thickened only flush to the outer surface (b), so that transverse costae were absent; this level continues into the irregular pattern (d) along the distal margin, which appeared to have no areolae: the spaces between the irregular borders appeared to lack areolae and were interpreted as pits. Striae in valve depression not clearly seen; Figure 9E (arrow) suggests areolae may be filled in along outer edge of valve depression. Internally a longitudinal break in striae along base of keel but there were areolae on the keel wall (Figure 9C arrowhead). Girdle bands (Figure 9A,B,F) with bordered areolae in multiple decussate rows, one (valvocopula?) with an abvalvar margin of bordered pits like that on valve; pleura with single line of bordered areolae and margin of bordered pits (Figure 9B).

Etymology: from the corrugated effect of the costae on the conopea and in the peri-raphe zone.

#### 3.1.9. *Homoeocladia ornata* Lobban, Sison and Ashworth, sp. nov.

Figure 10A–G

Diagnosis: Ribbed conopea and fully bordered areolae, differing from *H. corrugata* in the bordered areolae in the peri-raphe zone, uniformly bordered areolae on exposed valve–mantle face, and lack of pitted zone along valve border.

Holotype: Figure 10A–E, from specimen on stub 226, according to Article 40.5 of the International Code of Nomenclature for algae, fungi, and plants (Shenzhen Code) [41].

Registration: http://phycobank.org/104097

Type locality: PALAU: Babeldaob Island, Ngaremlengui State, dock at Bkulangriil, 07°31.488′ N, 134°29.966′ E, sand sample rich in *Carinasigma*, sample PW2009-46. Coll. 11 April 2009, C.S. Lobban and M. Schefter.

Morphology: Based on the holotype observed straight on and tilted 30°: valves lanceolate, rostrate, 15 µm long, 3 µm wide (Figure 10A–E). Striae parallel, 55 in 10 µm; fibulae not observed. Areolae quadrate, closed at the inner surface (Figure 10E). Areolae on exposed valve–mantle uniformly bordered, the virgae generally thickened more than the vimines, the distal ends of the striae with curved borders; areolae in peri-raphe zone with horse-collar shaped borders, extending to apex (Figure 10B–E). Glimpses under conopea (Figure 10D, arrow) suggest areolae may be infilled there. Conopea edges thickened and/or curled, surface with irregular longitudinal ridges (Figure 10B–D). Raphe raised between two ribs, one of which is noticeably thickened at the apices (Figure 10B,C). Valve face with short costae at apex, longer on one side (Figure 10B arrows) and a single costa extends along the peri-raphe zone above the bordered areolae (Figure 10D). Internal face of valve only partially seen, showing areolar occlusions flush with interior surface (Figure 10E). Copulae with up to six rows of uniformly bordered areolae; one band apparently a narrow pleura (Figure 10A, arrow). Figure 10F suggests that one of the bands in Guam material has striae with smaller areolae.

Etymology: L. *ornatus* = decorated.

Additional records: GUAM: Merizo Municipality, Achang mangrove, black mangrove pneumatophore. Specimens from GU58G-4A! and GU58G-4D! (Figure 10F,G); again, no internal view.

### 3.2. Theme 2: Large Linear Species

Three large linear species already described [35,37] all have quite different areolae external openings: in *H. asteropeae* (Lobban, Ashworth, Calaor and E.C.Theriot) Lobban and Ashworth they are oval pore fields, in *H. spathulatoides* (Lobban, Ashworth, Calaor and E.C.Theriot) Lobban and Ashworth they are small single pores, and in *H. tarangensis* (Lobban) Lobban and Ashworth they are transapically oval pores. The latter two species are spathulate, i.e., with a prominent crest on the keel near the apex. The pattern of pores in the peri-raphe zones also differs among the three species: in *H. asteropeae,* they are completely infilled, in *H. spathulatoides* there are short transapical rows of pores uniformly along both sides of the zone, and in *H. tarangensis* one longitudinal row on one side of the raphe and two to three on the other. We found another spathulate species with still different areolae external openings, along with several species differing from *H. tarangensis* in the number and distribution of other pores and areolae, and some lacking the keel crest (Table 2).

#### 3.2.1. *Homoeocladia asteropeae* (Lobban, Ashworth, Calaor and E.C.Theriot) Lobban and Ashworth

Figure 11A–C

**Figure 11:**
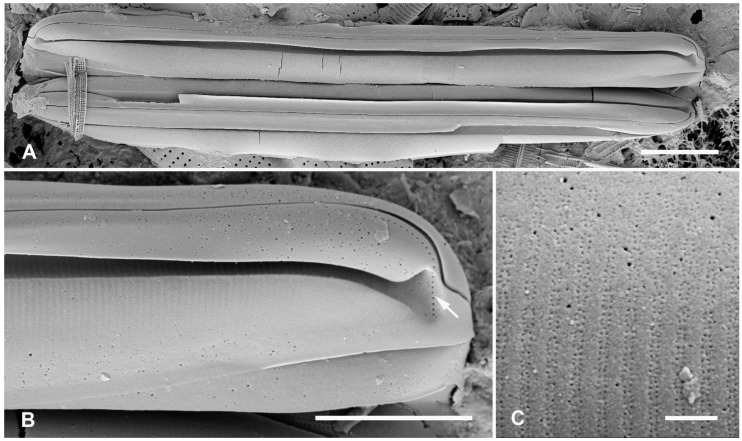
*Homoeocladia asteropeae* in SEM (sample GU52X-5). (**A**) Frustule from wild material. (**B**) Detail of apex in profile, showing line of small pores (arrow). (**C**) Detail to show areola structure. Scale bars: (**A**) = 10 µm, (**B**) = 5 µm, (**C**) = 500 nm.

This species was described primarily from a culture and here we provide some images of wild material. Conopea were rarely incised in the field, in contrast to the cultured cell line, and we no longer consider the incision of conopea to be a taxonomic character. We previously reported incised conopea in wild *H. guamensis* (Lobban, Ashworth, Calaor and E.C.Theriot) Lobban and Ashworth and an undescribed variation of *H. schefterae* (Lobban, Ashworth, Calaor and E.C.Theriot) Lobban and Ashworth. The profile (girdle) view (Figure 11B) shows the extent of the row of small pores on the apex, reaching the valve margin on the primary side (cf. Figure 16B). Image of valve exterior (Figure 11C) confirms the small oval pore fields in the areolae.

#### 3.2.2. *Homoeocladia ngesaolensis* Lobban, Sison and Ashworth, sp. nov.

**Figure 12:**
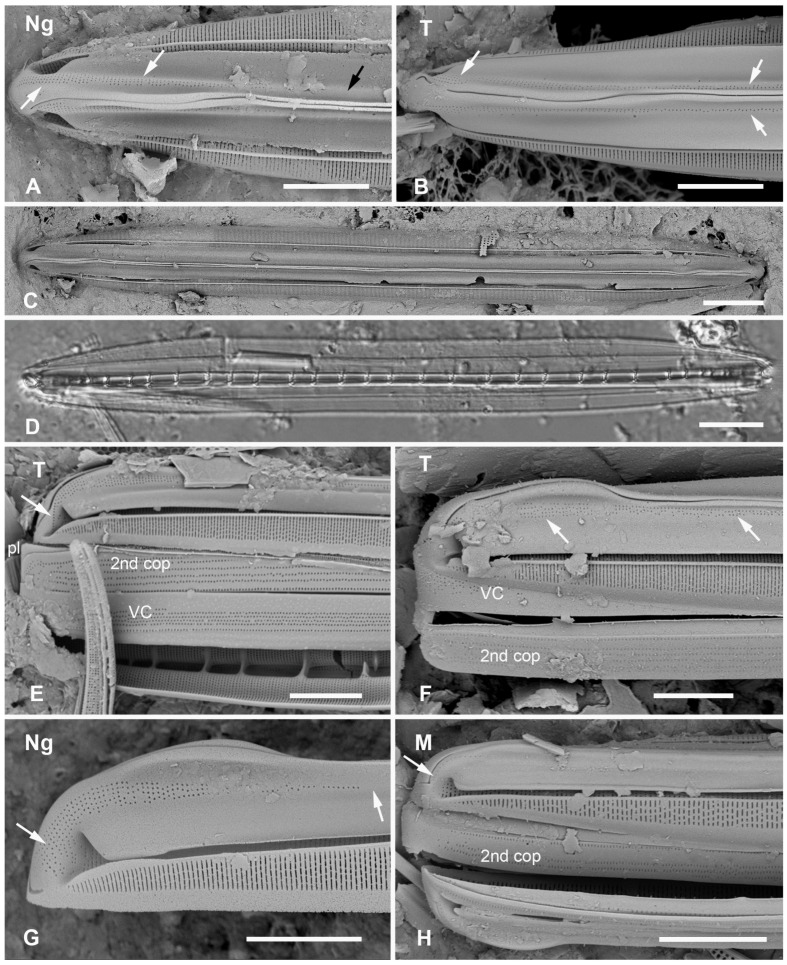
*Homoeocladia ngesaolensis* sp. nov. and comparisons with similar species. Since there are images of several species on this plate, we have annotated the comparisons with Ng (*H. ngesaolensis*), T (*H. tarangensis*), or M (*H. micronesica*). (**A**,**B**) Apex of holotype of *H. ngesaolensis* (**A**) showing short rows of pores on apex and below keel crest (white arrows), absent from rest of peri-raphe zone (black arrow), compared to *H. tarangensis* at same scale (**B**), showing pores continuing along peri-raphe zone in peri-raphe zone (white arrows). Both species have keel crest and costa along edge of valve depression. (See also comparison of profile views in (**E**–**H**).) (**C**) *H. ngesaolensis* entire holotype specimen in SEM. (**D**) LM of putative *H. ngesaolensis*, distinguished from *H. micronesica* in the same sample by the lower stria density. (**E**–**H**) *H. ngesaolensis* girdle views of apex in SEM compared to *H. tarangensis* and *H. micronesica* (not to same scale). (**E**,**F**) *H. tarangensis* showing broad girdle bands (VC, 2nd cop); pores continuing along peri-raphe zone (arrows (**F**)) plus a single line of pores at the apex (arrow (**E**)). [(**E**) shows a hypovalve plus epicingulum with pleura (pl), (**F**) shows a valve with its own cingulum.] (**G**) *H. ngesaolensis* showing pore distribution on apex and end of peri-raphe zone; arrows show differences from *H. tarangensis*. (**H**) *H. micronesica* showing single row of small pores on apex (arrow), absence of pores in peri-raphe zone, and areolae of 2nd copula. Scale bars: (**C**,**D**) = 10 µm, (**A**,**B**,**E**–**H**) = 5 µm.

**Figure 13:**
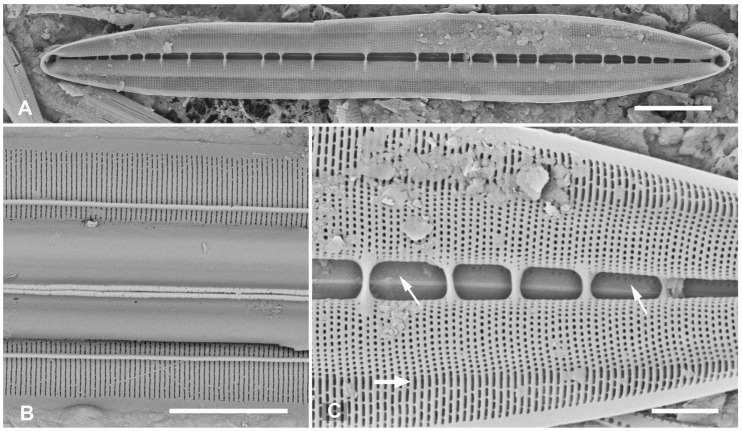
*Homoeocladia ngesaolensis* sp. nov. in SEM. (**A**) Valve in internal view. (**B**) External view in SEM showing costae on valve depression boundary and bordering the raphe. (**C**) Internal detail of keel canal toward apex showing pores near apex but not further away (regular arrows) and longer foramina under areolae along edge of valve depression (thick arrow). Scale bars: (**A**) = 10 µm, (**B**) = 5 µm, (**C**) = 2 µm.

Figure 12A,C,D,G and Figure 13A–C

Diagnosis: Large, linear valves with keel crest, costa along edge of valve depression, distinguished from *H. tarangensis* most readily by the absence of pores along the peri-raphe zone and absence of single row of pores on apex but also areola density and length of exposed striae.

Holotype: Figure 12A,C and Figure 13B from specimen on stub 1519, according to Article 40.5 of the International Code of Nomenclature for algae, fungi, and plants (Shenzhen Code) [41].

Registration: http://phycobank.org/104098

Type locality: PALAU: Koror, Ngesaol, north edge of mangrove, 07°21.268′ N, 134°30.310′ E, sample PW1990-47. Coll. January 1990, C.S. Lobban, M. Schefter, and D. Smith.

Morphology: Valves linear, tapering to rounded apices, with keel crest, 124–163 µm long, 12–13 µm wide; striae parallel, 46 in 10 µm; fibulae fairly uniformly spaced, 2 in 10 µm, except closer at the apices (Figure 12A,C,G and Figure 13A). Edge of conopeum and costa along edge of valve depression visible in LM (Figure 12D). Keel raised above conopea, raphe between a pair of costae (Figure 12A and Figure 13B). Mantle with a wide (ca. 1.6 µm) hyaline border (Figure 12G and Figure 13B,C). Areolae, 30–35 in 10 µm, opening by irregular short transapical slits on both sides of the exposed valve surface across this zone but externally recessed between virgae (Figure 13B,C) (striae 2.0 µm long, versus 1.4 µm in *H. tarengensis*), opening by circular pores under the valve depression (Figure 13B,C). Internally, a row of generally longer slits subtending the costa along edge of valve depression (Figure 13C). At apex, several areolae in each stria above conopea open by small pores, the number gradually diminishing below keel crest (Figure 12A,G), and pores absent from most of the peri-raphe zone, as seen also in internal details of keel (Figure 13C). No single row of pores on apex (Figure 12G; contrast *H. tarangensis*, Figure 12E).

Girdle bands not found with valve specimens. The comparison images of *H. tarangensis* in girdle view (Figure 12E,F) show its girdle bands, not previously observed, comprising a valvocopula with five rows of pores, the advalvar one slightly further from the other four, and a probably narrow pleura, only glimpsed (Figure 12E).

Etymology: Named for the type locality in Palau.

Additional records: PALAU: PW2022-1A-9! (Koror, Ngetmeduch mangrove, 7°21′29″ N, 134°29′50″ E, not far from type locality).

Comments: Scarce but not rare. Specimens were found in two nearby mangroves but collected nearly 30 years apart, suggesting that this is a stable species and not an aberrant population. The internal views into the keel confirm that the absence of pores along the peri-raphe zone is not a result of external closures making them invisible [compare Figure 15D with ([35], Figure 110)]. Co-occurring with *H. micronesica* sp. nov. in PW1990-47 but readily distinguished by fibula density (Table 2).

#### 3.2.3. *Homoeocladia micronesica* Lobban, Sison and Ashworth, sp. nov.

**Figure 14:**
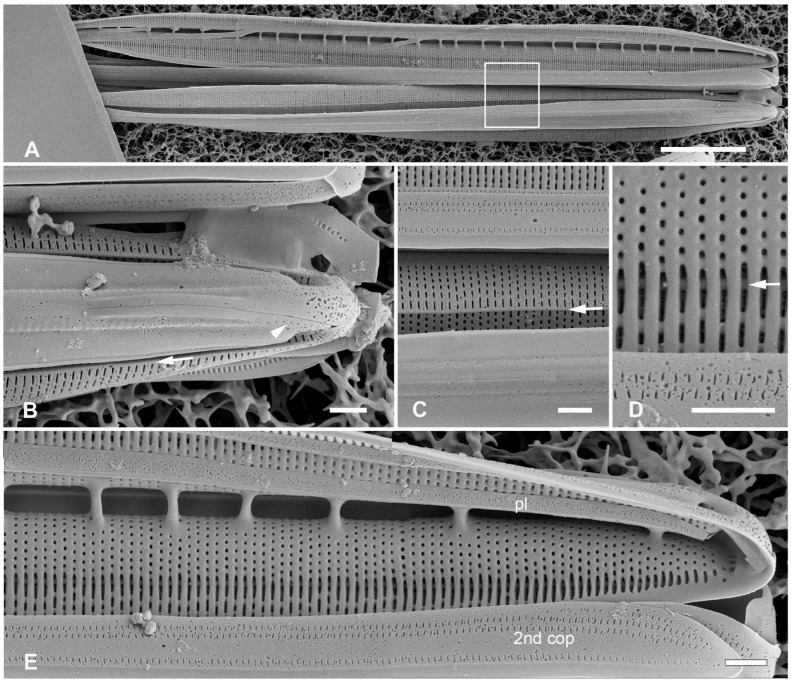
*Homoeocladia micronesica* sp. nov. holotype in SEM. (**A**) Entire frustule, framed area enlarged in C. (**B**) Apex showing keel crest, line of small pores (arrowhead), and costa along edge of valve depression (arrow). (**C**) Portion of frustule showing keel and conopea (bottom of image), external valve surface with costa along boundary of valve depression (middle of image) and putative valvocopula (VC) with one row of slits along the advalvar side, next to pars interior, and two rows on the abvalvar side. (**D**) Detail of internal infilling under exposed valve face (middle of image) and valve depression (top of image), also showing longitudinal costa (arrow) and part of external copula abvalvar surface. (**E**) Internal apex with girdle bands: open ends of 2nd copula and pleura (pl). Scale bars: (**A**) = 10 µm, (**B**–**E**) = 1 µm.

Figure 12H and 14A–E

Diagnosis: Large, linear valves with keel crest, boundary along edge of valve depression, distinguished from *H. tarangensis* most readily by the absence of pores along the peri-raphe zone and absence of single row of pores on apex but also areola density and length of exposed striae.

Holotype: Figure 14A–E, from specimen on stub 226, according to Article 40.5 of the International Code of Nomenclature for algae, fungi, and plants (Shenzhen Code) [41].

Registration: http://phycobank.org/104099

Type locality: PALAU: Babeldaob Island, Ngaremlengui State, dock at Bkulangriil, sand sample rich in *Carinasigma*, 07°31.488′ N, 134°29.966′ E, PW2009-46. Coll. 11 April 2009, C.S. Lobban and M. Schefter.

Morphology: Valves 86 µm long, µm wide, linear with cuneate apices and crested keel; raphe not raised between ribs (Figure 12H and Figure 14B,C). Fibulae 4 in 10 µm, striae parallel throughout, 44–46 in 10 µm (Figure 14C–E). Areolae transapical slits, tending to be longer along the edge of the valve depression, where a boundary line (not clearly a costa) separates the areolae in the valve depression (Figure 14B); the last exposed areola and first covered areola together subtended by a single elongated foramen (Figure 14D,E). Single row of small pores on each side of apex, no other pores or areolae opening in peri-raphe zone (Figure 12H and Figure 14B,C). Internal foramina circular under valve depression (Figure 14D). Girdle bands (Figure 12H and Figure 14E): valvocopula not clearly seen; 2nd copula, identified from Figure 12H, with three rows of slits in the pervalvar axis, two rows separated from each other by wide space; a narrow pleura, pitted but not clearly perforated (Figure 14E).

Etymology: Named for the region of origin, Micronesia, and in honor of *Micronesica*, the University of Guam’s journal of natural history of the region, which has a 60-year history of publishing biodiversity records.

Additional records: PALAU: PW1990-47!; FEDERATED STATES OF MICRONESIA: Yap, Y26C!

Comments: Co-occurring with *H. ngesaolensis* in PW1990-47 but readily distinguished by fibula density (Table 2).

#### 3.2.4. *Homoeocladia vittaelatae* Lobban, Sison and Ashworth, sp. nov.

Figure 15A–I

Diagnosis: Differing from *H. tarangensis* in lack of keel crest, higher fibula density and areola density, wide girdle bands with multiple rows of densely packed pores.

**Figure 15:**
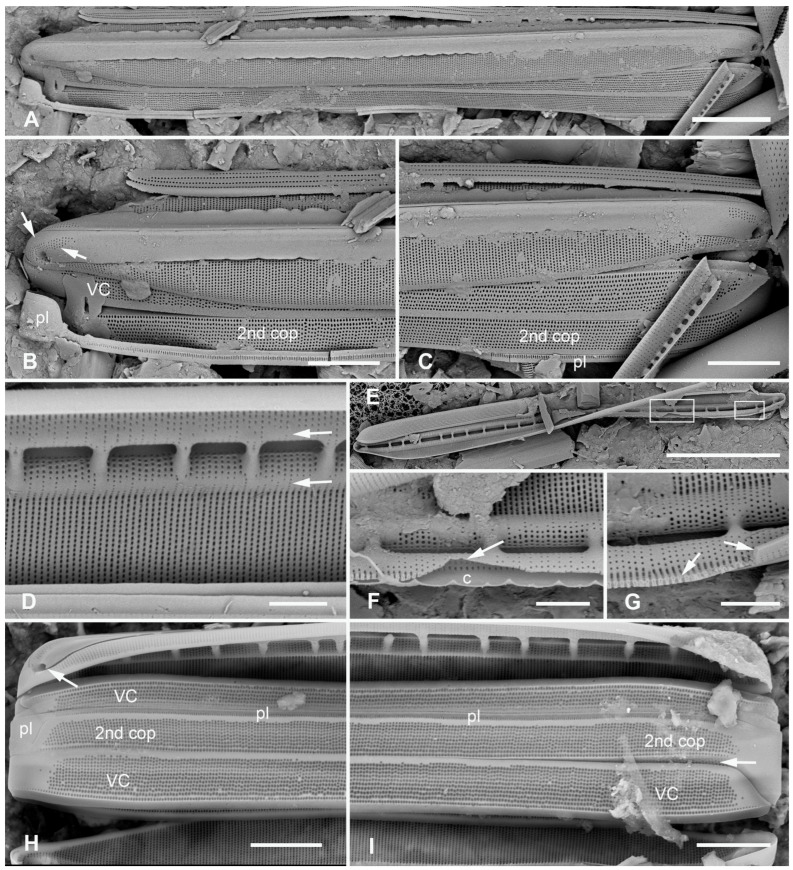
*Homoeocladia vittaelatae* sp. nov. in SEM (sample Y26C). (**A**–**C**) Holotype. (**A**) Entire frustule. (**B**,**C**) Details of the two apices, showing swarm of pores on apex but no line of small pores (arrows) and three girdle bands (labeled as before). (**D**) Interior valve surface with regular infilling but irregular break in striae crossed by occasional striae (arrows). (**E**–**G**) Specimen showing interior and valvocopula. (**E**) Whole specimen, frames mark details of broken edges enlarged in (**F**,**G**). (**F**) Undersurface of conopeum (c) and side of keel, along with difference in thickness of exposed valve wall vs. in conopeal canal. (**G**) Detail of cross-sectioned striae and margin (arrows). (**H**,**I**) Frustule in girdle view: details of poles showing girdle bands. Showing also the point on conopeum (arrowhead, (**H**)) and pores on pars interior of 2nd copula (arrow, (**I**))). Scale bars: (**E**) = 25 µm, (**A**) = 10 µm, (**B**,**C**,**H**,**I**) = 5 µm, (**D**,**F**,**G**) = 2 µm.

Holotype: Figure 15A–C, from specimen on stub 1350, according to Article 40.5 of the International Code of Nomenclature for algae, fungi, and plants (Shenzhen Code) [41].

Registration: http://phycobank.org/104100

Type locality: FEDERATED STATES OF MICRONESIA: Yap, Tarang (“O’Keefe’s Island”), 09°31.502′ N, 138°07.944′ E; subtidal sediments from 15 m depth, sample Y26C. Coll. 25 Sep. 1988, C.S. Lobban and M. Schefter.

Morphology: Valves 89–98 µm long, 11 µm wide, striae parallel, 47 in 10 µm, fibulae 3.5 in 10 µm. No keel crest, no areolae in peri-raphe zone (Figure 15A–C). Areolae small, transapically oval, usually closed at the surface (Figure 15D vs. Figure 15B,C), 60 in 10 µm. (vs. *H. tarangensis* ca. 40, *H. ngesaolensis* 30–35). Areolae showing above and beyond end of conopeum at apex, but lacking single line small pores (Figure 15B,C). Outer ends of conopea extend beyond the attachment point (Figure 15H). Internally, areolae of valve–mantle opening by regular oval foramina, those in the valve depression via circular openings; an unusual irregular longitudinal break along base of valve depression: some striae with continuous open foramina across the break (Figure 15D, arrows; Figure 15F,G). The exposed valve wall is much thicker than the wall under the conopeum (Figure 15F,G). Girdle bands (Figure 15H,I): Two broad copulae: a valvocopula, and a 2nd copula; the former closed at one end but tapering abruptly from the abvalvar side at the open end, with up to 12 rows of oval areolae in transverse striae; 2nd copula with a large ligula at the closed end, aligned with the open end of the valvocopula, which has up to 10 rows of pores in striae with these striae continuing onto the pars interior (Figure 15I). The narrow pleura has a large ligule fitting into the open end of the 2nd copula and a single row of short slits (Figure 15B,C,H,I).

Etymology: L. *vittae* (ribbons) + *latae* (broad), with reference to the broad copulae.

### 3.3. Theme 3: Areolae Opening by Sinuous Slits

**Figure 16:**
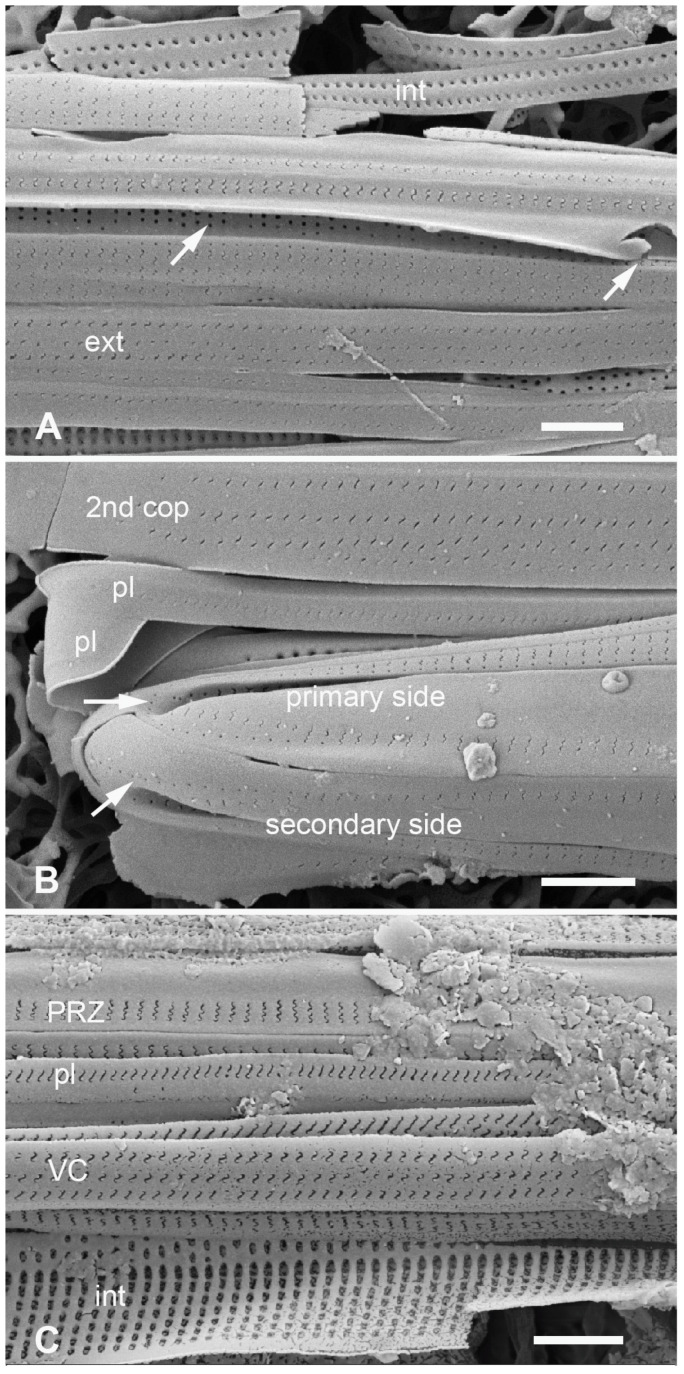
*Homoeocladia celaenoae* in SEM (specimen from PW2009-46). (**A**) Portion of frustule near apex showing pores in valve depression to be circular except sinuous at apex (arrows). Girdle bands in interior (int) and exterior (ext) view, showing that vela are at outer surface. (**B**) Apex of mature frustule with two pleurae (epi- and hypotheca), the open end of one lying on top of the closed end of the other. (**C**) Portion of a frustule with exterior surfaces of peri-raphe zone (PRZ), pleura (pl), and valvocopula (VC), interior of exposed valve surface (int), showing oval foramina. Scale bars all 1 µm.

Among the species previously described from Guam, *H. celaenoae* (Lobban, Ashworth, Calaor and E.C.Theriot) Lobban and Ashworth was unique in having sinuous external areolae openings. We found additional specimens in samples from Palau and they showed more clearly that most pores under conopeum are circular (sinuous only near the apex) (Figure 16A–C). Vela were at the outer side of the areolae, subtended by transapically oval openings, infilled to small ovoid pits under valve depression; likewise on the girdle bands (Figure 16A). In samples from Guam, Yap, and Palau, we also found specimens with sinuous openings but differing in other characters from *H. celaenoae*. One of those was also similar to *H. schefterae*, giving us the data we needed to address the question, left unanswered in 2019: “There were **2–3 rows of slits on the pleurae in the type specimen** (Figure 141)… but we have also observed a specimen from a different location in which there were **two sets of lineate striae** on one of the pleurae, and **another where the slits were S-shaped**.… The variation in [girdle band] perforations may indicate finer separation of taxa but we do not have the data to assess this further” [35] (p. 230, emphasis added). Closer views of the type specimen are shown below in Figure 24.

#### 3.3.1. *Homoeocladia sinuosa* Lobban, Sison and Ashworth, sp. nov.

Figure 17A–H

Diagnosis: Valves with sinuous external areola openings but differing from *H. celaenoae* especially the presence of keel crest, infilled pores in the peri-raphe zone, and linear–lanceolate outline rather than lanceolate.

Holotype: Figure 17A–E, from specimen on stub 1475, according to Article 40.5 of the International Code of Nomenclature for algae, fungi, and plants (Shenzhen Code) [41].

Registration: http://phycobank.org/104101

Type locality: GUAM: Outhouse Beach, Apra Harbor, 13°27.840′ N, 144°39.360′ E, biofilm over calcareous sand, ca. 8 m deep, sample GU52X-5. Coll. 10 May 2015, C.S. Lobban and M. Schefter.

Morphology: Valves linear–lanceolate with a keel crest, 109–116 µm long, 12 µm wide, striae 47 in 10 µm (Figure 17A,D,F), fibulae not observed. Areolae on valve face and in valve depression opening by short sinuous slits (~) (Figure 17C,E), boundary of valve depression marked by hyaline line (Figure 17C,E) but lacking row of adjacent small pores (cf. *H. celaenoae*). On the apex, a short band of rows of three areolae with small circular openings, the most medial equal to the single rows of apical pores in other species (Figure 17B,E); the band of openings abruptly terminated at the apical ends of the keel crests, the pores in the peri-raphe zone between those points infilled, leaving faint lines (ridges?) (Figure 17D,E). Internal valve only glimpsed (Figure 17G), no longitudinal break in striae under valve depression. Areolae on girdle bands with sinuous external openings as in valve (Figure 17E); one band with five rows of pores (Figure 17G).

Etymology: Named for the short, sinuous external openings of the valve face areolae and girdle bands.

Comments: If our interpretation of the peri-raphe zone is correct, it makes this species the only one in the group without areolae there (Table 2).

#### 3.3.2. *Homoeocladia interrupta* Lobban, Sison and Ashworth, sp. nov.

Figure 18A–G

Diagnosis: Areolae opening via curly slits on valve, star-shaped slits on girdle bands; side of keel crest ribbed; wide hyaline break in areolae at outer edge of valve depression.

Holotype: Figure 18A–D, from specimen on stub 226, according to Article 40.5 of the International Code of Nomenclature for algae, fungi, and plants (Shenzhen Code) [41].

Registration: http://phycobank.org/104102

Type locality: PALAU: Babeldaob Island, Ngaremlengui State, dock at Bkulangriil, sand sample rich in *Carinasigma*, 07°31.488′ N, 134°29.966′ E, PW2009-46. Coll. 11 April 2009, C.S. Lobban and M. Schefter.

Morphology: Valves lanceolate–linear, 69 µm long, 6 µm wide; striae parallel, 41 in 10 µm; fibulae 4–5 in 10 µm (Figure 18A,G). Keel crest with one or two horizontal ridges on the side (Figure 18C,F). Prominent longitudinal costa along edge of valve depression (Figure 18B,D). Areolae on exposed valve face sometimes completely infilled, otherwise opening by irregularly curly slits (Figure 18D) (not simple sinuous slits as in *H. sinuosa*). Areolae in peri-raphe zone completely filled in; line of small pores on each side of the apex (Figure 18B). Internally, transapically-oval foramina under the exposed valve face, circular foramina under valve depression, striae interrupted in a wide band near outer edge of depression (Figure 18E,F). Girdle bands: two copulae with x- or star-shaped slits in the areolae, pores in decussate pattern, valvocopula wider than 2nd copula; narrow pleurae with one and two rows (Figure 18B,C).

Etymology: Named for the unusually placed internal break in the striae and the sporadic breaks in striae on the exposed valve–mantle face.

Comments: The longitudinal break has an unusual location along the outer part of the depression, rather than at the base of the keel [cf. *H. ngiwalensis* Figure 7C and *H. volvendirostrata* (Ashworth, Dąbek and Witkowski) Lobban and Ashworth ([35], Figure 24). Besides the diagnostic characters, it is distinguished from *H. celaenoae* also in lower stria density and from *H. sinuosa* in smaller size and a costa along the boundary of the valve depression.

#### 3.3.3. *Homoeocladia irregularis* Lobban, Sison and Ashworth, sp. nov.

Figure 19A–D

Diagnosis: Valves lanceolate, differing from *H. celaenoae* in having irregularly infilled areolae on valve face and a prominent keel crest; differing from *H. sinuosa* in having a row of small pores along edge of valve depression and open sinuous areolae in peri-raphe zone, and from *H. interrupta* in lacking a longitudinal break in striae.

Holotype: Figure 19A–D, from specimen on stub 226, according to Article 40.5 of the International Code of Nomenclature for algae, fungi, and plants (Shenzhen Code) [41].

Registration: http://phycobank.org/104103

Type locality: PALAU: Babeldaob Island, Ngaremlengui State, dock at Bkulangriil, sand sample rich in *Carinasigma*, 07°31.488′ N, 134°29.966′ E, PW2009-46. Coll. 11 April 2009, C.S. Lobban and M. Schefter.

Morphology: Valve lanceolate with spathulate keel, ca. 60 µm long, 5 µm wide, fibulae 4–5 in 10 µm, striae parallel, 55 in 10 µm (Figure 19A,C). Areolae with sinuous external openings, but irregularly infilled internally (Figure 19C,D); single row of long sinuous areolae openings in peri-raphe zone (Figure 19C); row of small pores on the apex (Figure 19B). Internal foramina transapically elongate ovals under exposed valve–mantle face; a row of single circular pores along the edge of the valve depression, opening in larger circular foramina; smaller circular foramina in the valve depression; infilling around the bases of most fibulae (Figure 19A,C,D). External surface only glimpsed at apex (Figure 19B); girdle bands not seen.

Etymology: Named for the irregular infilling of the internal valve face.

Comments: Although this was seen in a single internal view, the sinuous character of the areolae was visible and clearly links it to other species in this group, while the combination of other character states clearly distinguishes it from them. There appears to be some sort of boundary at the edge of the valve depression (Figure 19C,D, arrows) but it is between foramina, rather than showing as an external costa or break visible through the foramen as in *H. dagmannii* ([35], Figure 103).

#### 3.3.4. *Homoeocladia celaenopsis* Lobban, Sison and Ashworth, sp. nov.

Figure 20A–G

Diagnosis: *Homoeocladia* with S-shaped areola openings, differing from *H. celaenoae* in having two rows of areolae along the peri-raphe zone, a hyaline line along edge of valve depression but no line of circular pores, sinuous openings also in the valve depression, and a weakly elevated keel crest.

Holotype: Figure 20A–C, from specimen on stub 1573, according to Article 40.5 of the International Code of Nomenclature for algae, fungi, and plants (Shenzhen Code) [41].

Registration: http://phycobank.org/104104

Type locality: PALAU: Babeldaob Island, Ngiwal State, Lekes mangrove, 07°31.867′ N, 134°37.183′ E, in mud scraped from red mangrove pneumatophores, sample PW2021-4-7. Coll. 7 July 2021, Kebang Ngeraklang, Palau Community College.

Morphology: Valves lanceolate, very weakly spathulate, 46–58 µm long, 6 µm wide, striae parallel, 50 in 10 µm (Figure 20A–F). Areolae with strongly sinuous openings (Z-shaped) on exposed valve–mantle surface and under conopea (Figure 20B–E), interrupted externally by an apparently flat hyaline line (Figure 20C–E). Two rows of areolae in the peri-raphe zone, also with Z-shaped slits, and a single line of small circular pores on each side of the apex (Figure 20B,D). Internally (as seen in Yap specimens, Figure 20F,G), fibula density 3–4 in 10 µm; foramina oval under exposed valve–mantle face, circular under valve depression, a line of longer foramina under the edge of the depression (Figure 20G (arrow)). No break in striae at base of keel. Girdle bands: two copulae with 3 and 4 rows of mostly diagonal, slightly sinuous slits and a pleura with one row (Figure 20F,G).

Etymology: Named for its similarity to *H. celaenoae*.

Additional material: FEDERATED STATES OF MICRONESIA: Yap: Y26-C!, Tareng (O’Keefe’s Island), in sediments from sponge, ca. 8 m depth.

#### 3.3.5. *Homoeocladia schefteropsis* Lobban, Sison and Ashworth, sp. nov.

Figure 21A–H and Figure 22

Diagnosis: Lanceolate valves with long slits in peri-raphe zone, break along edge of valve depression; areolae on exposed valve–mantle opening by straight or irregular slits, differing from *H. schefterae* in areolae on valve and girdle bands having S-shaped external openings and in having a costa on the external edge of the valve depression.

Holotype: Figure 21A–D, from specimen on stub 226, according to Article 40.5 of the International Code of Nomenclature for algae, fungi, and plants (Shenzhen Code) [41].

Registration: http://phycobank.org/104105

Type locality: PALAU: Babeldaob Island, Ngaremlengui State, dock at Bkulangriil, sand sample rich in *Carinasigma*, 07°31.488′ N, 134°29.966′ E, sample PW2009-46. Coll. 11 April 2009, C.S. Lobban and M. Schefter.

Morphology: Lanceolate valve 37–40 µm long, 4.5 µm wide, fibulae 5 in 10 µm, striae 55–56 in 10 µm (Figure 21A,E). Areolae opening on exposed valve–mantle via transapical slits varying in length and often wavy (Figure 21B,C), under the valve depression interior openings smaller, circular (Figure 21D,F,G). Boundary line (not clearly a costa) interrupting the striae along edge of valve depression, with small pore in each stria on the distal side; internally, forked areola openings on the depression side (Figure 21D,F). No internal break in striae under depression (Figure 21E,G). Areolae opening along peri-raphe zone by long slits, sometimes with small fork at the end, continuing along the keel crest to mantle (Figure 21B–D); additional row of small circular pores on each side of the apex (Figure 21C,F). Girdle bands with three rows of areolae with distinctly sinuous (~), often diagonal external slits and circular internal foramina, rows on copula evenly spaced, those on valvocopula with one further from the other two; pleura with one row of pores (Figure 21C–E,H and Figure 22).

Etymology: Named for its resemblance to *H. schefterae*, particularly in the peri-raphe zone areolae.

Comment: Distinguished from *H. celaenoae* by the keel crest and valve areolae, and from *H. schefterae* (Figure 24) by the keel crest and sinuous areolae. The holotype is the specimen that was alluded to in our 2019 paper [35], quoted above.

Additional records: PALAU: Ngiwal, PW2021-4-7!; FEDERATED STATES OF MICRONESIA: Yap, Y26C!; U.S.A. Guam: Outhouse Beach, Apra Harbor, GU52K-7!

#### 3.3.6. *Homoeocladia coacervata* Lobban, Sison and Ashworth, sp. nov.

Figure 23A–F

Synonym: *Nitzschia schefterae* Lobban, Ashworth, Calaor and E.C.Theriot 2019 ([35], in part: Figures 7 and 144).

Diagnosis: Differing from *H. schefterae* in the stacks of apically oriented lineate areolae on the copulae.

**Figure 23:**
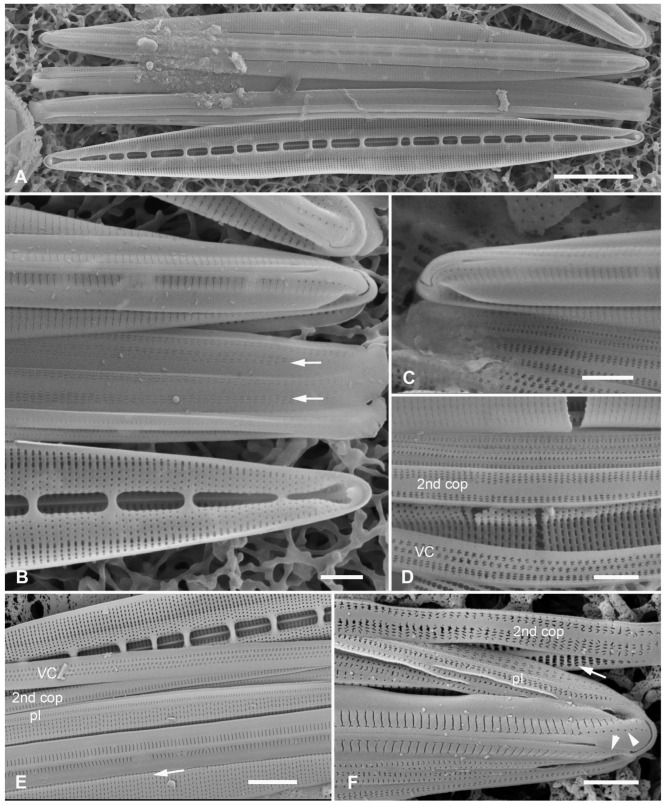
*Homoeocladia coacervata* sp. nov. in SEM. (**A**,**B**) Holotype (GU52K-7). Entire frustule and enlargement of one pole, the latter showing internal and external valve faces and girdle bands with stacked slits (arrows). (**C**,**D**) Portions of a Guam frustule (same specimen as shown in ([35], Figure 7)), showing pattern of slits on copulae. (**E**) Portion of frustule from Majuro (M6-31) showing valve faces and girdle bands. Note absence of internal longitudinal break in striae at base of keel; lack of boundary at edge of valve depression (arrow). (**F**) Majuro specimen (M2-10) showing detail of apex with slits in peri-raphe zone and row of small pores on apex, with internal and external sides of 2nd copula, arrow showing a set of slits is within one oblong foramen. Scale bars: (**A**) = 5 µm, (**E**) = 2 µm, (**B**–**D**,**F**) = 1 µm.

Holotype: Figure 23A,B, from specimen on stub 230, according to Article 40.5 of the International Code of Nomenclature for algae, fungi, and plants (Shenzhen Code) [41].

Registration: http://phycobank.org/104106

Type locality: U.S.A. Guam: Outhouse Beach, Apra Harbor, 13°27.840′ N, 144°39.360′ E, farmer fish turf (*Stegastes nigricans*), ca. 5 m deep, sample GU52K-7. Coll. 3 May 2009, C.S. Lobban and M. Schefter.

Morphology: Valves lanceolate, 30–39 µm long, 3–4 µm wide, fibulae 6–7 in 10 µm, striae parallel, 55 in 10 µm (Figure 23A). Single line of areolae in the peri-raphe zone opening by long slits, continuing to the end of the conopeum, below a line of small pores on each side of the apex (Figure 23B,C,F). Areolae on exposed valve–mantle face opening by straight transapical slits except for a single circular pore in each stria at the edge of valve depression; no boundary along this edge (Figure 23E arrow and Figure 23F). Internal foramina oval under valve face, circular under the valve depression; no break in striae at base of keel (Figure 23B,E). Girdle bands with stacks of horizontal slits (i.e., along apical plane *vice* pervalvar plane) (Figure 23B–F): valvocopula with three evenly spaced rows of stacks, 2nd copula with two unequal stacks on opposite sides of a wide hyaline stripe, the slits open in a common foramen (Figure 23F arrow); narrower pleura with single row of stacks.

Etymology: L. from *coacervare*, to pile up, accumulate, with reference to the stacked slits in the areolae on girdle bands.

Additional records: Guam: GU21AQ!, GU44BF-1A!; PALAU: PW2009-22!, PW2009-46!; FEDERATED STATES OF MICRONESIA: Yap, Y26C!; MARSHALL ISLANDS: Majuro, M2-10!, M2-13!, M6-31!

Comments: This species is not part of the “sinuous areola openings” group, of course, but is treated here to complete the revision of *H. schefterae*. It was the co-dominant *Homoeocladia* in the Majuro samples along with *H. dagmannii*.

## 4. Discussion

Many additional *Homoeocladia* species were found both within samples and between Micronesian islands, while several species occurred on several islands. Almost all were rare and mostly in samples with relatively abundant species such as *H. dagmannii,* which is apparently commonly found across Micronesia (Appendix A). This is the typical pattern for microbes [42] (p. 813): “Biological communities are normally composed of a few abundant and many rare species. This pattern is particularly prominent in microbial communities, in which most constituent taxa are usually extremely rare”. Samples with high *Homoeocladia* diversity included PW2009-46 (sand biofilm; 10 species), M2-13 (filamentous algae; 6 species), GU52K-7 (farmer fish algal turf; 5 species), PW2021-4-7 (mangrove mud; 4 species). Samples with few but new *Homoeocladia* species included PN2-9 (mangrove mud), GU66F-8 (farmer fish algal turf), and GU52X-5 (biofilm) (Appendix A).

Seventeen new species were grouped on shared characters, i.e., (1) any form of external thickenings, (2) large, linear valves, and (3) sinuous external slits in the areolae. The first group had additional shared characters, especially large areolae, which might indicate a common ancestor, but we do not expect that “large, linear valve outline” is a meaningful phylogenetic character combination, because size and shape are often variable even within species of diatoms, and we know of large lanceolate species. (Our “large” species here are >80 µm, including two species previously described [35]). In earlier work [35] there was congruence between clades in the molecular tree and the morphological tree, as far as sequence data were available, especially a group with *H. schefterae* + *H. celaenoae*. However, the sequenced materials of “*H. schefterae*” (HK464 and HK465) are now both identified as *H. coacervata* (see Supplemental Figures S1 and S2) and, of the three morphological synapomorphies that defined that clade, two are now shown to belong to *H. schefteropsis*, i.e., “advalvar row VC areolae S-shaped” and “pleurae areolae S-shaped”. Thus, we are not suggesting monophyly for any of the morphogroups under discussion.

The potential biodiversity in this genus is likely largely undescribed, as continued collections have led to no sense of nearing an asymptote in taxon discovery. For example, we have yet to describe our imaged Micronesian specimens in the group with lanceolate valves lacking external thickenings. Almost all the new species were rare and found only after preparation for SEM. We recognize the limitation of not having molecular data for any of them, but we did not anticipate nearly so many species. Adequate taxon sampling for culturing and DNA analysis would be a large new project.

### 4.1. Morphological Characters

Girdle bands within and between species showed a variety of patterns in the arrangement of pores, especially the numbers and spacing of longitudinal rows alignment of transverse or decussate rows, and these potentially have taxonomic value when the bands can be identified. Two problems confounded the identification of bands in wild material: (1) frustules tended to fall in valve view and collapse such that bands overlapped; (2) isolated bands could not be assigned to species. Nevertheless, girdle bands were identified in many species and were differentiated into two broader copulae, often with different numbers of rows of pores, and usually one narrow pleura with one row or none. Bands often had similar areolae to the exposed valve surface, but this was not always so, as seen in *H. interrupta* and *H. coacervata.* In the context of species flocks, the ancestral state might be “girdle-band areolae match valve areolae”, as little variation in girdle-band areolae patterning has been documented in *Bacillaria* and *Gomphotheca*, two putative sister taxa for *Homoeocladia*, though diversity in these genera are still relatively low compared to *Homoeocladia* and *Nitzschia* sensu lato. Nonetheless, differentiation within girdle-band areolae might then indicate a species flock. As with *Licmophora* species [33,43], when girdle bands do have characters that differ among species, these characters should be documented to the extent possible.

Apical rows of small pores occur in some species and may continue to the mantle or not ([35], Figures 19 and 20). This needs some clarification however, as the two lines follow different paths: ([35], Figure 8) and here in Figure 16B and Figure 17B,E. The terminal raphe fissures bend in the same direction, which is normally to the secondary side [44]. The raphe branch deflects slightly to one side before hooking to the other, so that the row on the secondary side is inside the hook and the line on the primary side is outside. Thus, in girdle view, the one on the primary side may go to the mantle or not (depending on species) but on the secondary it always remains on the keel and would be said not to reach the margin. Since only one side can be seen in girdle view, it is easy to mistake the character state. In the 2019 examples [35], all the views are of the row outside the terminal fissure, both valves in each image oriented the same way. In *H. meropeae* (Lobban, Ashworth, Calaor and E.C.Theriot) Lobban and Ashworth, valve-face striae occur between the row of pores and the margin, in *H. dagmannii*, they do not. The primary-side row is often evident in internal views (e.g., Figure 21F, *H. schefteropsis*). Since the terminal raphe fissures bend in the same direction (see Figure 11A, *H. asteropeae*), the appearance of the apical row of pores (if any) in girdle view is the same at both ends. Apical rows were absent in the large-areolae species of the “bordered areolae” theme; *H. radiata* (Figure 4B,D) had apical rows but also differed in having small areolae.

Costae: It was often difficult to judge the comparative thickness of the virgae or vimenes relative to the basal silica layer, but the close-up view of *H. corrugata* (Figure 9E) is helpful because relative height can be judged, while in *H. equitorquis* the smooth valve face (Figure 8E) implies it is the surface of the basal silica layer. We had initially assumed that any thickenings on the outside were costae, based on experience with the earlier species, in which external costae were generally longitudinal (“ribs”) that sometimes cut across areolae as an external bar visible in internal views (*H. dagmannii*, ([35], Figure 103)). We decided to categorize such continuous longitudinal or transverse thickenings as costae, categorizing other situations, such as the “horse collars” around the peri-raphe zone areolae in *H. equitorquis*, as bordered areolae (Table 2). Nevertheless, it was unclear in *H. micronesica* and *H. schefteropsis* whether the boundary of the valve depression was flat or raised as a costa; consequently, we defined only two states for the boundary character, present/absent (Table 2). *Homoeocladia interrupta* had a clear costa in that position but it was not distinguishable from internal views because of the unusual location of the longitudinal break in striae (Figure 18D,E).

There was often a ridge on the valve border at the apex (e.g., Figure 8B and Figure 10G), which, in some cases, continued as the costa along the edge of the valve depression; this was clearly the case in *H. tarangensis, H. ngesaolensis* (Figure 12A,G), *H. micronesica, H. celaenoae,* and *H. celaenopsis*, but we also see it in *H. jordanii* (Figure 5), where the opposite slopes of the valve on each side of this costa confirm that the valve depression did not follow the reduction in the conopeum width, in contrast to *H. ngiwalensis*, in which the ridge continued along the edge of *the valve* (Figure 6B arrowheads), with more costae medially and the valve depressions narrowing along with the conopea (Figure 6E). In other species, the apical ridge ended (often unequally on the two sides: Figure 10B), either not connecting to a boundary of the valve depression, or when there was no boundary [*H. ornata* (Figure 10B,G), *H. equitorquis* (Figure 8A,H), respectively]. Some species, e.g., *H. asteropeae* and *H. vittaelatae*, had neither an apical ridge nor a valve depression boundary. We could not determine the situation in all species from our image bank.

### 4.2. Comparisons within Morphological Groups

*External thickenings*. Species with this theme mostly shared quadrate areolae and the presence of external costae and/or bordered areolae. Shapes and dimensions of these species were very similar (Table 2), mostly 15–30 µm long, the only exception being *H. radiata*, which was up to 50 µm long and had small areolae. The putative ancestral state, with no external thickenings but with similar large areolae, is represented by *H. alcyoneae* and *H. marshallensis*. The areolae of *Homoeocladia marshallensis*, *H. majurana,* and *H. ngiwalensis* had hymenes at the outer surface, in contrast to *H. equitorquis, H. corrugate,* and *H. ornata*, where hymenes were at the inner surface (Figure 2H, Figure 3C and Figure 6E vs. Figure 8E, Figure 9F and Figure 10E). Pitted valve borders occurred on *H. contraria* and *H. corrugata* (Figure 7F and Figure 9E).

*Large linear valves:* We selected large linear valve morphology as a theme because our initial survey of *Homoeocladia* from Yap samples gave glimpses of several species close to *H. tarangensis*, the one we were already able to describe [37]. In addition, *H. spathulatoides* and *H. asteropeae* have this theme. As noted in the Results, the areolae of the known species vary widely, but areolae in the new species—*H. ngesaolensis*, *H. micronesica,* and *H. vittaelatae*—were all like those of *H. tarangensis*. Only the latter two were from Yap, however; *H. ngesaolensis* has been found so far only in two samples from Palau, nearby locations but 30 years apart. Besides the areolae, no other characters link the three new species either to *H. tarangensis* or to all the group: the presence of keel crests, costae along the edges of the valve depression, and distribution of pores in the peri-raphe zone and on the apex differed with no pattern apparent yet.

*Sinuous areolae:* Sinuous areolae occurred in only one of the species reported in 2019 [35], *H. celaenoae*, so their presence in five additional species was surprising. The species with this theme largely shared the following additional characters (Table 2): a boundary along the external edge of the valve depression, the presence of areolae in the peri-raphe zone, and an apical row of small pores on the keel. While these shared characters might suggest a common ancestor for the group, the fact that one of them is otherwise similar to *H. schefterae* suggests that sinuous areolae may not be a synapomorphy for this group, i.e., that the apparent morphological clade may be paraphyletic.

### 4.3. The Homoeocladia schefterae Question

**Figure 24:**
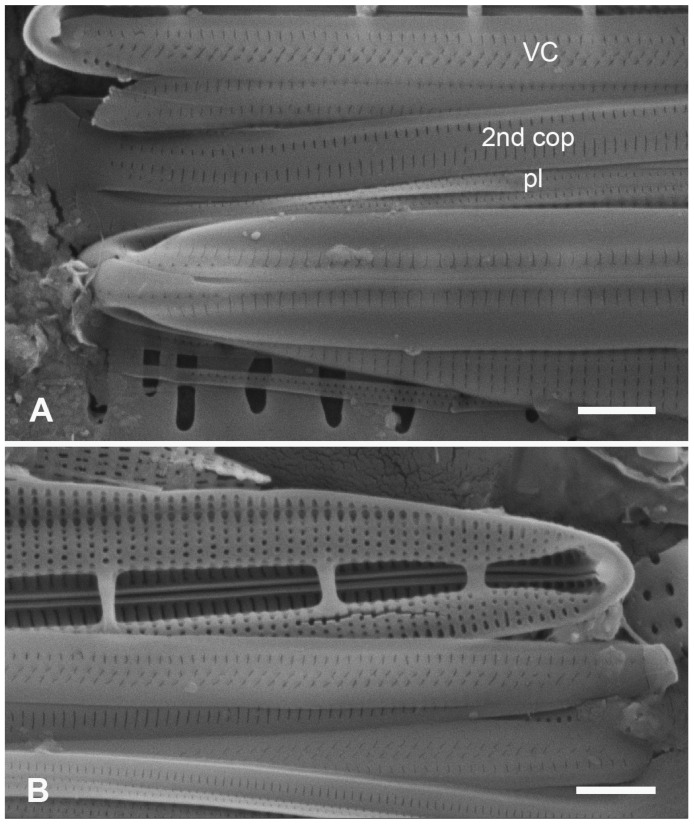
(**A**,**B**) *Homoeocladia schefterae* in SEM (GU52K-7). Details of holotype specimen (entire frustule shown in ([35], Figure 141)). Note single linear slits on copulae, in contrast to *H. coacervata*. Scale bars = 1 µm.

**Figure 1:**
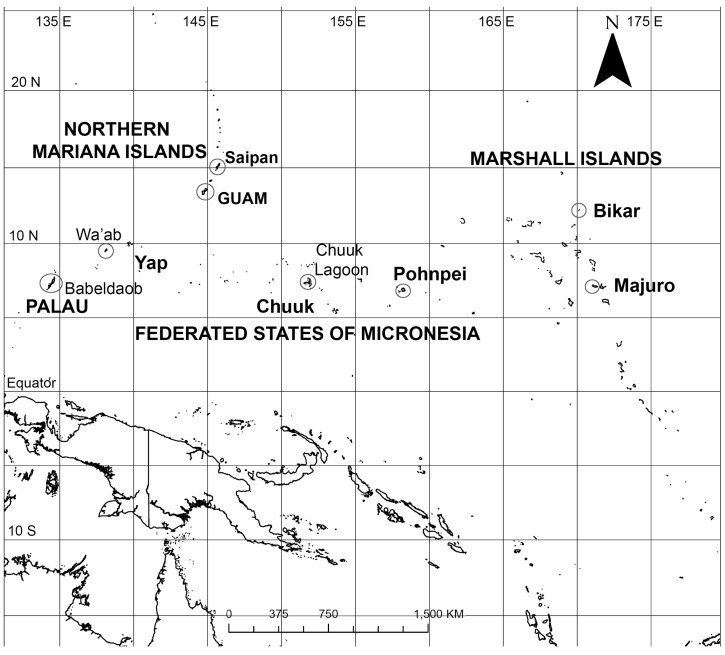
Map of Micronesia in relation to New Guinea and Australia, showing the collection sites for the *Homoeocladia* project.

**Figure 6:**
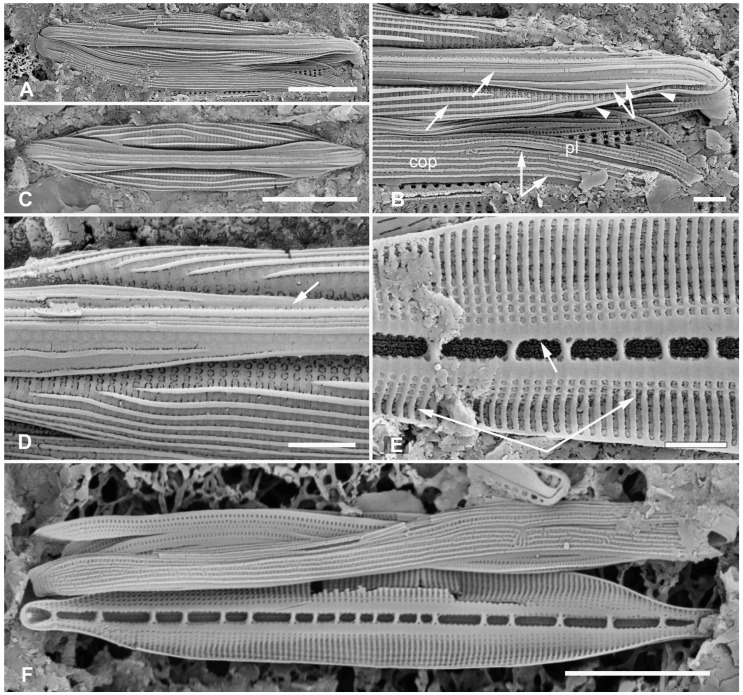
*Homoeocladia ngiwalensis* sp. nov. in SEM (sample PW2021-4-7). (**A**,**B**) Holotype: whole valve with girdle bands and detail of apex showing costae (arrows from left to right): on valve face along the vimines; alongside the raphe; beside rows of areolae on the girdle bands; and on conopeum. Arrowhead shows the apical ridge becoming a costa on the edge of the valve rather than the edge of the valve depression. (**C**) Isolated valve emphasizing the radiating costae on valve face but striae parallel. (**D**) Specimen with striae radiate toward apex. (**E**,**F**) Valve interior with girdle bands and detail of internal valve surface showing edge of depression approaching keel in the middle (joined arrows, (**E**)) and longitudinal break in striae at base of keel. Costae, rather than just infilled basal layer, are suggested by the ridges evident across the hyaline zone and seen in profile at edge of keel (arrow, (**E**)). Scale bars: (**A**,**C**,**F**) = 5 µm, (**B**,**D**,**E**) = 1 µm.

**Figure 8:**
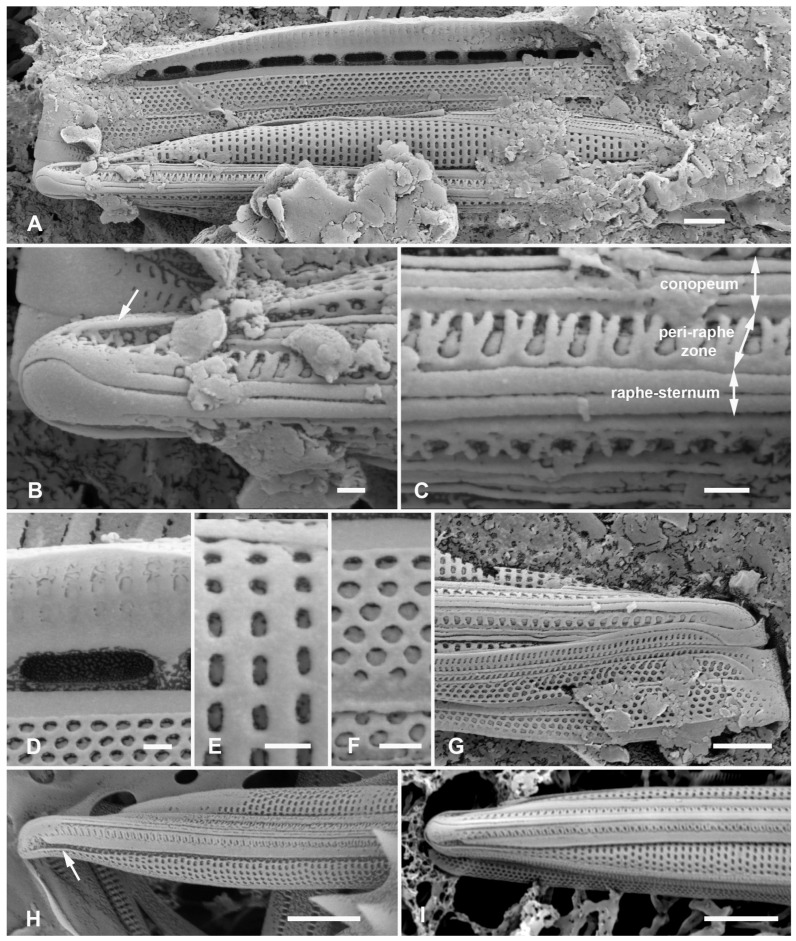
*Homoeocladia equitorquis* sp. nov. in SEM. (**A**–**F**). Holotype from PW2021-4-7. (**A**) Entire frustule. (**B**) Detail of apex showing costa near apex (arrow) that does not continue along edge of valve depression. (**C**) High magnification of central portion of keel and conopea, showing the bordered areolae in the peri-raphe zone (PRZ) and costae bordering the raphe and on the conopea. (**D**) Detail of internal surface showing break in longitudinal striae and vela of areolae at inner side of wall. (**E**,**F**) Details of areolae on exposed valve face and a copula seen from exterior, again showing vela at inner side of wall. (**G**) Specimen showing girdle bands. (**H**,**I**) Two specimens from Jellyfish Lake, Palau, lacking costae on the conopea but showing the short costa on the valve apex (arrow, (**H**)). Scale bars: (**H**,**I**) = 2 µm, (**A**,**G**) = 1 µm, (**B**–**F**) = 200 nm.

**Figure 9:**
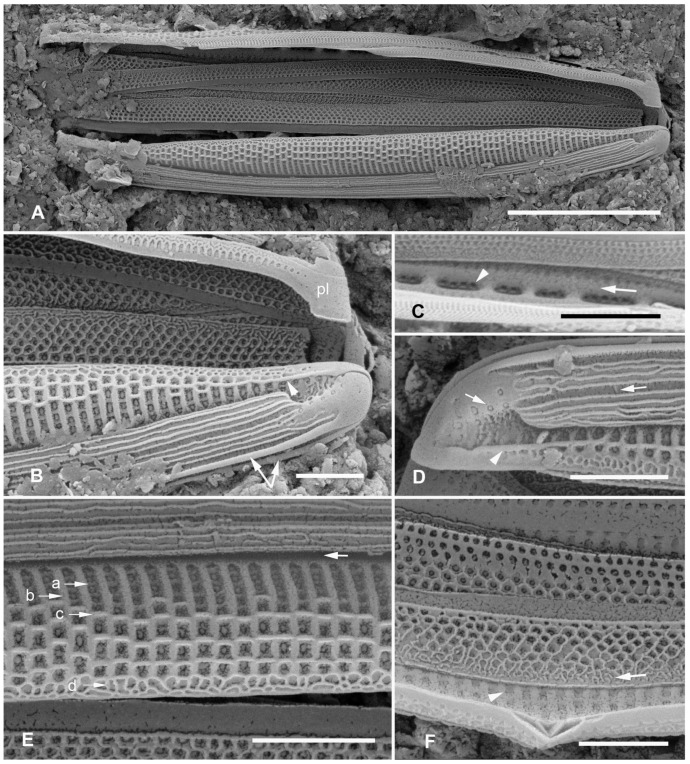
*Homoeocladia corrugata* sp. nov. in SEM (sample PN2-9). (**A**–**C**) Holotype. (**A**) Entire frustule. (**B**) Detail of apex showing corrugation both on conopea and in peri-raphe zone, along with ribs bordering raphe (double arrow) and short apical costa (arrowhead) continuous with first thickened vimines. Three copulae visible (probably two valvocopulae and one 2nd copula) along with pleura (pl). (**C**) Detail of inner surface showing break in longitudinal striae (arrow) and pores in peri-raphe zone from inside (arrowhead), not clear between costae on outside (**D**) Detail of different apex showing more irregular corrugation; putative areolae in the peri-raphe zone indicated by thick arrow, line of unbordered areolae to its right (arrows) and short apical costa (arrowhead). (**E**) Details of thickenings on exposed valve surface: (a) ordinary vimen; (b) thickened virga; (c) thickened vimines; (d) irregular thickening around pits. Unlabeled arrow in shows that external costae do not continue into the conopeal canal. (**F**) Detail of frustule showing girdle bands and glimpse of interior valve surface. Note especially pitted border (arrow) on one copula (valvocopula?), resembling border on valve, and position of vela relative to inner surface (arrowhead). Scale bars: (**A**) = 5 µm, (**C**) = 2 µm, (**B**,**D**–**F**) = 1 µm.

**Figure 10:**
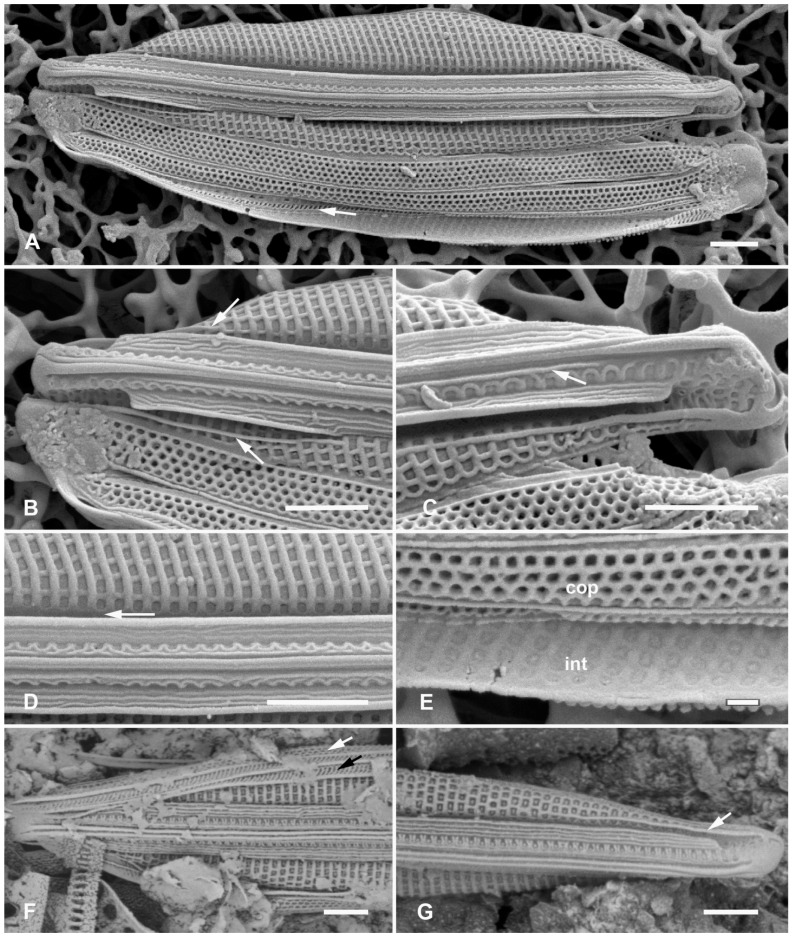
*Homoeocladia ornata* sp. nov. in SEM. (**A**–**E**) Holotype frustule from PW2009-46. (**A**) Whole frustule, arrow indicates narrow pleura. (**B**) Detail of apex (no tilt), showing short costae on valve at apex (arrows), bordered areolae on exposed valve face, in peri-raphe zone, and on copulae; arrow indicates narrow pleura. (**C**) Detail of opposite apex, tilted 30°, showing corrugated conopea, short apical costa, thin costa in peri-raphe zone (arrow). (**D**) Detail of middle portion of frustule showing external valve and keel with view into conopeal canal showing thickenings absent (arrow). (**E**) Detail from mid portion of frustule showing outer part of internal valve surface (int), and external portion of copula (cop). (**F**,**G**) Specimens from Guam; note contrasting areolae in the copulae ((**F**), white vs. black arrows) and apical costa on valve face ((**G**) arrow). Scale bars: (**A**–**D**,**F**,**G**) = 1 µm, (**E**) = 200 nm.

**Figure 17:**
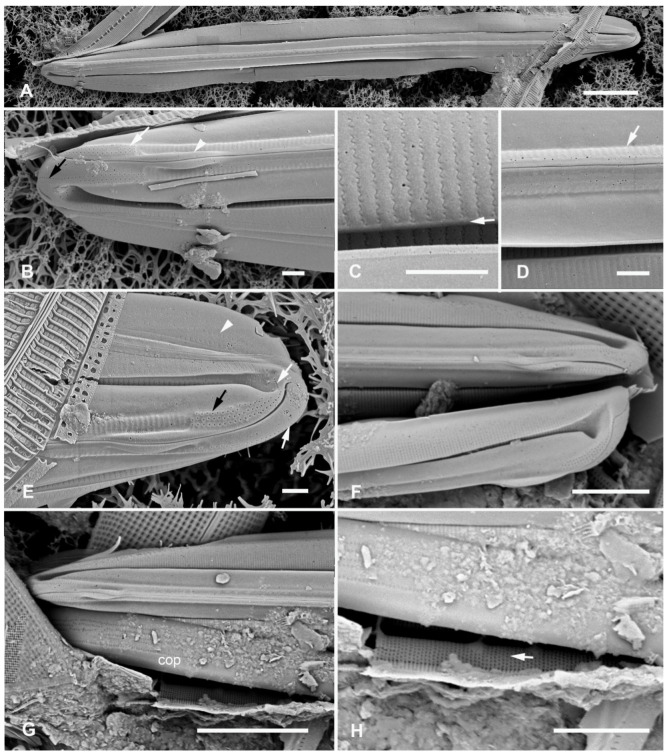
*Homoeocladia sinuosa* sp. nov. in SEM (sample GU52X-5). (**A**–**E**) Holotype. (**A**) Entire valve. (**B**) Apex showing prominent keel crest (arrowhead), band of pores between keel crest and end of conopeum (white arrow) and single line of pores on apex (black arrow). (**C**) Detail of valve surface with sinuous areola slits, including in valve depression, and hyaline line along edge of valve depression with no small pore in each stria (arrow). (**D**) Detail of keel and conopeum showing faint elevations in peri-raphe zone where the areolae are infilled (arrow). (**E**) Apex in oblique view showing three lines of pores ending at keel crest (black arrow), medial lines of small pores continuing to apex (white arrows), and the areolae on the copulae (arrowhead). (**F**) Apex of frustule showing valves in valve and girdle view with pattern of apical pores. (**G**,**H**) Opposite apex of same frustule with glimpse of internal surface and girdle bands: (**G**) contrast adjusted for pore pattern on copula to show five lines of pores, (**H**) greater magnification and contrast adjusted for exposed inner surface. Note absence of longitudinal break in striae at base of keel (arrow). Scale bars: (**A**,**G**) = 10 µm, (**F**,**H**) = 5 µm, (**B**–**E**) = 1 µm.

**Figure 18:**
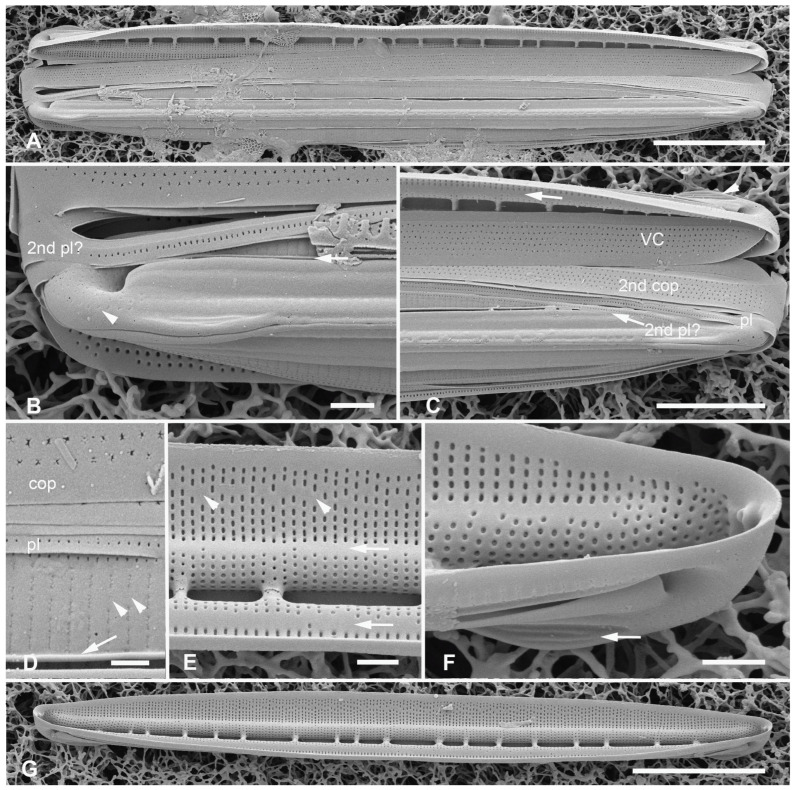
*Homoeocladia interrupta* sp. nov. in SEM (sample PW2009-46). (**A**–**C**) Holotype. (**A**) Entire frustule. (**B**) Apex of specimen showing apical pores (arrowhead); arrow points boundary of valve depression. (**C**) Detail of opposite apex showing apex in valve view, part of internal valve face with break in striae (arrow), and all three girdle bands with second pleura. (**D**) External detail showing irregularly curly slits of to costa along areolae and single-areola interruptions in striae (arrowheads); also visible at bottom of image is costa along edge of valve depression (arrow), and edge of conopeum; at the top, overlapping girdle bands including a copula and pleura, note x- or star-shaped openings on copula. (**E**) Portion of isolated valve in internal view, showing longitudinal break in striae at outer edge of valve depression (arrow) and single-areola interruptions in striae (arrowheads). (**F**) Isolated valve, detail of keel crest with ridges (arrow). (**G**) Entire valve in internal view. Scale bars: (**A**,**G**) = 10 µm, (**C**) = 5 µm, (**B**,**E**,**F**) = 1 µm, (**D**) = 500 nm.

**Figure 19:**
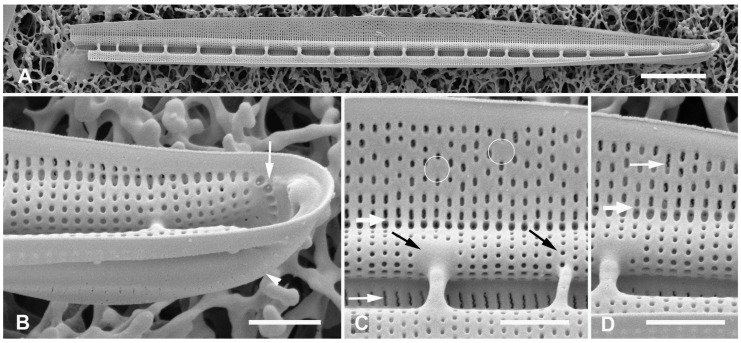
*Homoeocladia irregularis* sp. nov. holotype in SEM (sample PW2009-46). (**A**) Entire specimen, internal view. (**B**) Detail of apex showing keel crest and line of pores internally on primary side (arrow) and externally on secondary side (arrowhead). (**C**,**D**) Details showing sinuous external areola openings in peri-raphe zone and on valve face (white arrows on (**C**), (**D**), respectively), irregular infilling of areolae in the exposed valve face (circled), inconsistent interruption of striae around attachment of fibulae (black arrows), evidence of an external boundary at edge of valve depression, indicated by a line (thick arrows) between small pores with deep foramina and oval pits where the sinuous external areola openings are visible. Scale bars: (**A**) = 10 µm, (**B**–**D**) = 1 µm.

**Figure 20:**
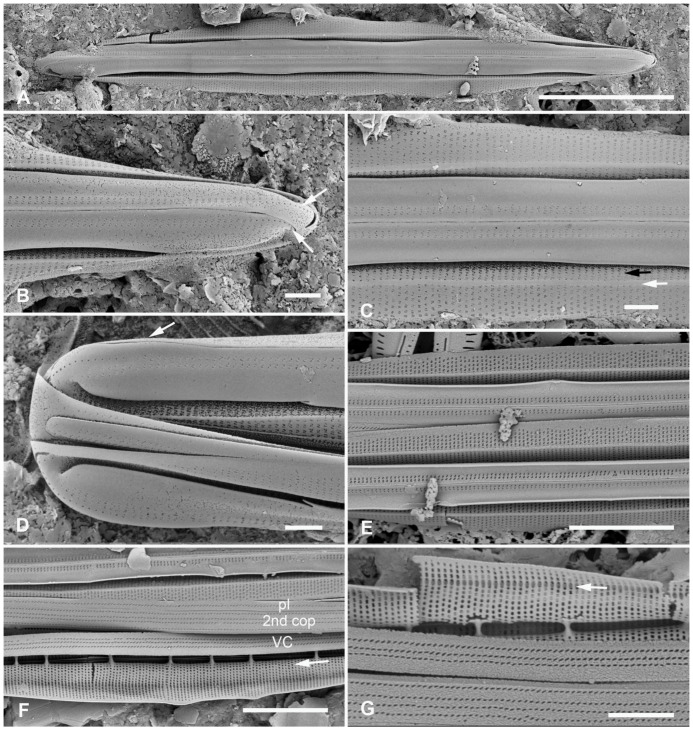
*Homoeocladia celaenopsis* sp. nov. in SEM (**A**–**D**), specimens from Palau, PW2021-4-7; (**E**–**G**) from Yap, Y26C. (**A**–**C**) Holotype valve. (**A**) Entire valve. (**B**) Apex showing sinuous areolae openings in peri-raphe zone and single lines of small pores on primary and secondary sides (arrows). (**C**) Mid portion of valve showing continuation of sinuous slits into the valve depression (black arrow), hyaline boundary along edge of depression (white arrow), and two rows of areolae in the peri-raphe zone. (**D**) Frustule in profile showing low rise in keel near apex (arrow). (**E**) Mid portion of frustule showing two valves in external view. (**F**) Mid portion of frustule showing inner and outer valve faces, inner showing absence of longitudinal break in striae at base of keel (arrow); also showing three girdle bands, tentatively identified relative to the pleura. (**G**) Detail of inner surface showing position of hyaline line at edge of valve depression (arrow). Scale bars: (**A**) = 10 µm, (**E**,**F**) = 5 µm, (**G**) = 2 µm, (**B**–**D**) = 1 µm.

**Figure 21:**
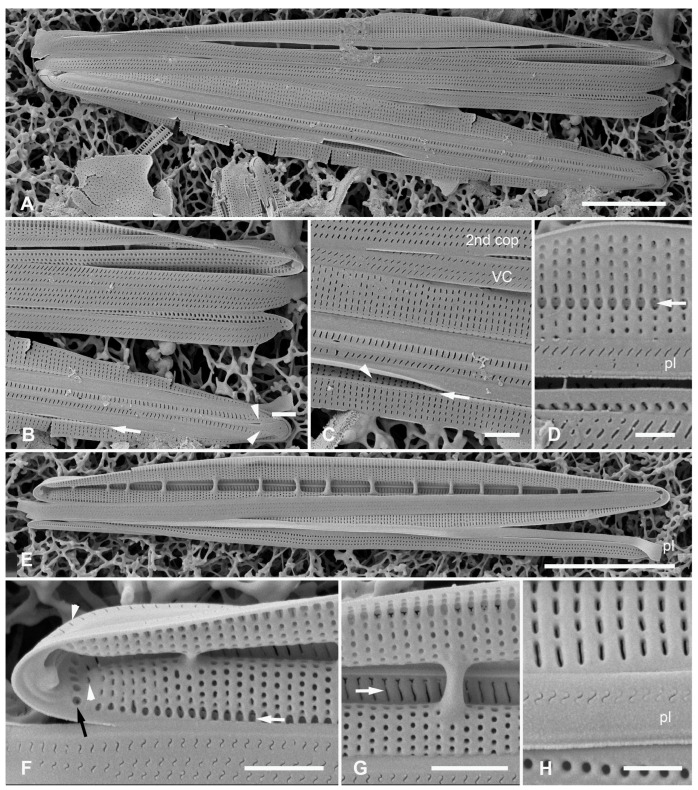
*Homoeocladia schefteropsis* sp. nov. in SEM (sample PW2009-46). (**A**–**D**) Holotype frustule. (**A**) Whole frustule. (**B**) Apex in valve view showing rows of small pores on apex (arrowheads), costa along edge of valve depression (arrow) and sinuous slits in girdle bands. (**C**) Detail of valve showing sinuous slits in exposed valve surface; row of small pores and costa at valve depression boundary (arrow), and branched slit inside boundary (arrowhead). (**D**) Interior valve surface showing costa, small pore, and forked slit within single internal foramen (arrow). (**E**–**H**) Specimen with internal view. (**E**) Entire specimen. (**F**) Detail of apex showing line of small pores (black arrow) and continuation of areolae from peri-raphe zone seen externally and internally (arrowheads), also showing the internal aspect of boundary as in (**C**) (white arrow). (**G**) Detail near apex showing slits in the peri-raphe zone (arrow) and absence of longitudinal break in striae. (**H**) Details of internal foramina in valve wall, also showing exterior of pleura and interior of a copula. Scale bars: (**A**,**E**) = 5 µm, (**B**,**C**,**F**,**G**) = 1 µm, (**D**,**H**) = 500 nm.

**Figure 22:**
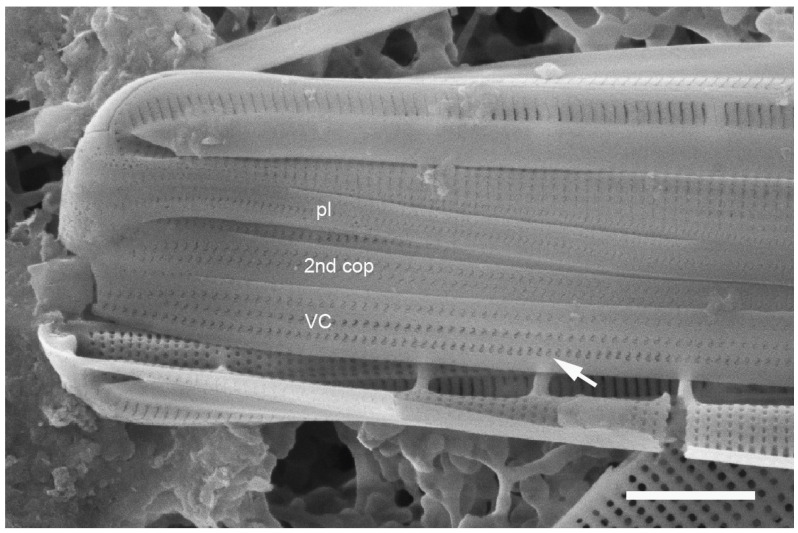
*Homoeocladia schefteropsis* sp. nov. Guam voucher specimen (GU44BF-1A), showing sinuous areolae (arrow). Scale bar = 2 µm.

Variation was noted in *H. schefterae* [35], but with additional specimens it has been possible to resolve the complex into so far three species. Details of the holotype specimen are shown in Figure 24 because the areolar detail in the printed image of the whole specimen ([35], Figure 141) is hard to see and ([35], Figure 144) is a different specimen (and species). *Homoeocladia schefteropsis* differed in having sinuous areolae on both valve and girdle bands, whereas *H. coacervata* differed only in the girdle bands. Since the type was defined on an SEM specimen in which the girdle band characters are visible, there is no ambiguity and new names were required for the others. As noted above, the vouchers of the two sequenced strains of “*H. schefterae*” reported in [35] turned out to be *H. coacervata*, and with one of those cultures derived from Florida (voucher specimens shown in Supplemental Figures S1 and S2), it is this new species that is widespread across Micronesia and into the Atlantic; *H. schefteropsis* and *H. schefterae* are less common and perhaps less widespread, though a culture of *H. schefterae* has been grown from an Iranian sample (Ashworth, unpubl.). Too few localities have been adequately sampled to define the biogeography of any of these species. Nevertheless, we have a potentially “cosmopolitan” ancestor (*H. coacervata*, present in Florida) with several potentially derived species in Micronesia. *Homoeocladia volvendirostrata* also seems to be widespread, known from Mozambique, Saudi Arabia, Mexico, Guam, and possibly temperate China [35,45,46].

## 5. Conclusions: Assessment of Potential for Species Flocks in *Homoeocladia*

We have now described 30 species of *Homoeocladia* from Micronesia and documented many more, establishing that this genus in this region meets the species richness criterion for species flocks. Five species described from other regions were transferred to *Homoeocladia* [36]; only one has been found so far in Micronesia [35]. While the genus appears to be monophyletic in our trees [35], low taxon sampling precludes any conclusions about monophyly within subflocks, and the character themes chosen here may well be misleading in this regard, as noted at the beginning of the Discussion.

The formation of species flocks requires the evolution of endemic species through adaptive or nonadaptive speciation and thus the existence of endemic species is a *sine qua non*. Fine-grained studies of diatom species have begun to show regional differences in diatom floras [31], e.g., between Arctic and Antarctic freshwater *Stauroneis* taxa [47,48]. A fine-grained study of *Licmophora* [33] presented evidence for different floras in Guam and Australia versus Europe, based on 6 new species out of 9 collected in Australia and 20 of 23 identified in Guam, even though all previous records of *Licmophora* in Australia had been attributed to known species. We concur with Vanormelingen et al. [31] (p. 395) that, “many diatom species contain several subtly distinct, semi-cryptic entities that are worth taxonomic recognition at the species level”. Our studies of genera in the Guam flora have repeatedly revealed such differences—not always subtle but overlooked nevertheless—and we have concluded [49] that Williams’ [26] provocative proposal, that everything is endemic, is a better starting point because, even if it is wrong, it will lead us to look more critically at taxa in our floras than an assumption based on “everything is everywhere”, i.e., that if a specimen seems to match a picture in the literature (mostly based on light microscopy), that must be it. We have been drawn to *Licmophora hyalina* as a “poster child” for this problem since it is typically identified by the absence of visible detail on most of the valve [49].

Much work is still needed on this genus. Intensive taxon sampling in Micronesia would increase the number of sequenced morphotypes and likely find new morphotypes. For the biogeographic questions, two approaches may be more readily achievable. First, a morphological survey with taxon sampling in Florida, where we are already conducting benthic diatom collections on a scale similar to the Micronesia effort, would tell whether rich biodiversity in this genus is found only in the Western Pacific, or wherever the genus occurs; and, if the latter, whether the species present are largely the same or not. Secondly, metabarcoding might show the diversity of taxa in different locations, albeit without morphological identities.

## Figures and Tables

**Table 1:** Collection data.

Entity	Sample Number	Location	Coordinates (N, E)	Date	Collector(s)
PALAU	PW2021-4-7	Babeldaob Island, Ngiwal State, Lekes mangrove	07°31.867′, 134°37.183′	7 July 2021	K. Ngeraklang
	PW2009-22 and -23	Jellyfish Lake	07°09.141′, 134°20.905′	9 April 2009	C.S. Lobban and M. Schefter
	PW2009-46	Babeldaob Island, Ngaremlengui State, Bkulangriil	07°31.488′, 134°29.966′	July 2021	K. Ngeraklang
	PW1990-47	Koror State, Ngesaol, north edge of mangrove	07°21.268′, 134°30.310′	January 1990	C.S. Lobban, M. Schefter and D. Smith
	PW2022-	Koror State, Ngetmeduch mangrove	07°21.483’, 134°29.833’	May 2022	R. Moreno and Y. Chibata
YAP (FSM)	Y26C	Wa’ab, Yap, Tarang (“O’Keefe’s Island”)	09°31.502′, 138°07.944′	25 September 1988	C.S. Lobban and M. Schefter
GUAM	GU52K-7	Piti Municipality, Apra Harbor, Outhouse Beach	13°27.840′, 144°39.360′	3 May 2009	C.S. Lobban and M. Schefter
	GU52X-5	Piti Municipality, Apra Harbor, Outhouse Beach	13°27.840′, 144°39.360′	10 May 2015	C.S. Lobban and M. Schefter
	GU68D-1B	Piti Municipality, Apra Harbor, Western Shoals	13°27.054′, 144°39.336′	12 August 2018	C.S. Lobban and M. Schefter
	GU58G-4A and -4D	Merizo Municipality, Achang mangrove	13°21.403′, 144°38.480′	13 April 2021	G. Prelosky, B. Sison and C.S. Lobban
	GU21AQ	Inarajan Municipality, Saluglula Pools	13°16.290′, 144°44.873′	19 December 2018	C.S. Lobban and M. Schefter
POHNPEI (FSM)	PN2-9	Kitti Municipality, Pehleng, Dauen Nahnsakar mangrove	06°52.767′, 158°09.390′	17 June 2021	M. L. Ling and B. Lynch
MAJURO (RMI)	M2-13, and M2-10	Majuro Atoll, Laura, lagoonside beach	07°09.530′, 171°02.336′	ca. 30 May 2022	V. Boktok
	M6-31	Majuro Atoll, Rita, lagoonside beach	07°07.337′, 171°21.664′	ca. 30 May 2022	V. Boktok

**Table 2:** Comparison of metrics and character states.

Species	Length, µm	Width, µm	Stria Density in 10 µm	Fibula Density in 10 µm	Areolae Character, Exposed Valve ^a^	Areolae in Peri-Raphe Zone	Boundary along External Edge of V.D. ^b^	Costae on ext. Valve (Besides Edge of V.D.) ^c^	Costae Bordering Raphe	Costae on Conopea and/or PRZ ^d^	Apices ^e^	Apical Row of Small Pores	Spathulate Keel (=Keel Crest Present)	Internal longit. Break in Striae Base of Keel	Internal Silicification Pattern ^f^	Thickenings on Girdle Bands ^g^
*Homoeocladia alcyoneae*	48–49	3	56	6–8	3	Y	N	0	N	0	0	Y	N	N	0	0
“Bordered areolae”																
*Homoeocladia marshallensis* sp. nov.	20–24	3	49–50	9	3	Y	N	0	N	0	1	N	N	N	2	0
*Homoeocladia majurana* sp. nov.	19	3	54	8	3	Y	Y	0	N	0	1	N	N	Y	0	0
*Homoeocladia radiata* sp. nov.	39–51	6	50	U	3	Y	N	0	Y	4	1	Y	Y	U	0?	1
*Homoeocladia jordanii*	15–16	3	51–58	10	3	Y	Y	1	Y	3	1	N	N	Y	0	1
*Homoeocladia ngiwalensis* sp. nov.	24	4–5	52	10	3	Y	N	1	Y	4	1	N	N	Y	2	1
*Homoeocladia contraria* sp. nov.	24–31	5–6	50	9–10	3	Y	N	2	Y	0	1	N	N	Y	0	2
*Homoeocladia equitorquis* sp. nov.	17–24	4	50–52	10	3	Y	N	0	Y	1	1	N	N	Y	0	0
*Homoeocladia corrugata* sp. nov.	18–20	4	52	10	3	Y	N	2	Y	3	1	N	N	Y	0?	2
*Homoeocladia ornata* sp. nov.	15–16	3	55	U	3	Y	N	2	Y	1	1	N	N	U	0?	2
Large, linear valves																
*Homoeocladia spathulatoides*	110–125	11	45	2–4	8	Y	N	0	Y	0	1	Y	Y	N	0	0
*Homoeocladia asteropeae*	82–118	8–11	45–50	3–4	7	N	N	0	N	0	0	Y	N	N	0	0
*Homoeocladia tarangensis*	108–194	10–12	46	2	1	Y	Y	0	Y	0	1	Y	Y	N	0	0
*Homoeocladia ngesaolensis* sp. nov.	124–163	13	46	2	1	N	Y	0	Y	0	0	Y	Y	N	0	0
*Homoeocladia micronesica* sp. nov.	86	c. 7	44–46	4	1	N	N	0	N	0	0	Y	Y	N	1	0
*Homoeocladia vittaelatae* sp. nov.	86–98	11	47	3.5	1	N	N	0	N	0	0	N	N	Y	0	0
Sinuous areola openings																
*Homoeocladia celaenoae*	27–70	4–6	50–60	5	4	Y	Y	0	0	0	0	Y	N	N	0	0
*Homoeocladia sinuosa* sp. nov.	109–116	12	47	U	4	N	Y	0	N	0	0	Y	Y	N	U	U
*Homoeocladia interrupta* sp. nov.	69	6	41	4–5	4	Y	Y	0	N	0	0	Y	Y	Y	1	0
*Homoeocladia irregularis* sp. nov.	60	5	55	4–5	4	Y	U	U	U	0	0	Y	Y	N	1	U
*Homoeocladia celaenopsis* sp. nov.	46–58	6	50	3–4	4	Y	Y	0	N	0	0	Y	N	N	0	0
*Homoeocladia schefteropsis* sp. nov.	37–40	4.5	55–56	5	4	Y	Y	0	N	0	0	Y	N	N	0	0
*Homoeocladia coacervata* sp. nov.	30–39	3–4	55	6–7	2	Y	N	0	N	0	0	Y	N	N	0	0

^a^ Coding for areolae character exposed valve: (1) small circular/oval pores; (2) transapical slits; (3) larger, occluded pores; (4) S-shaped slit; (5) longitudinal slits (6) continuous cribrum field; (7) small pore fields; (8) minute pores. ^b^ Coding for boundary along edge of valve depression (VD): (N) absent, hyaline line or (Y) longitudinal costa present; (U) unknown. ^c^ Coding for costae on valve: (0) absent or low ridges, (1) multiple longitudinal costae; (2) transverse costae. ^d^ Coding for costae on conopea/PRZ: (0) neither; (1) conopea only; (2) PRZ only; (3) both; (4) at apex only. ^e^ Coding for apices: (0) acute/obtuse; (1) rostrate; (2) capitate. ^f^ Coding for internal silicification pattern: (0) Uniform/regular; (1) irregular; (2) *H. taygeteae* pattern. ^g^ Coding for thickenings on girdle bands: (0) none; (1) longitudinal; (2) bordered areolae.

## Data Availability

All images supporting this study are in an image bank maintained by the senior author (CSL). Copies of published and unpublished images of species can be provided on any reasonable request. Images taken at UT by MPA are also retained there.

## Supplementary Materials

The following supporting information can be downloaded at: https://www.mdpi.com/article/10.3390/plants12234073/s1, Figures S1 and S2. *Homoeocladia coacervata* voucher specimens from sequenced cultures. Apices of disrupted frustules from Guam and Florida showing internal and external valve surfaces and external copula surfaces, to show stacked slits on copulae (arrows). Figure S1. GU52X1_thinNitzC28 = HK465, Guam. Figure S2. 19X15_1B_thinraphidB11 = HK464, Florida. Scale bars = 1 µm.

## Appendix A

Species lists for the samples studied here (bold = common, underlined = type locality of new species), not including unnamed species. Listed west to east: Palau (PW), Yap (Y), Guam (GU), Pohnpei (PN), and Majuro (M). Collection sites listed in Table 1.

PW1990-47: ***H. dagmannii***, *H. schefterae*, *H. ngesaolensis* sp. nov., *H. micronesica* sp. nov.

PW2009-46: ***H. dagmannii***, *H. schefterae*, *H. celaenoae*, *H. ornata* sp. nov., *H. micronesica* sp. nov., *H. interrupta* sp. nov., *H. schefteropsis* sp. nov., *H. coacervata* sp. nov., *H. contraria* sp. nov., *H. irregularis* sp. nov.

PW2021-4-7: *H. ngiwalensis* sp. nov., *H. equitorquis* sp. nov., *H. celaenopsis* sp. nov., *H. contraria* sp. nov.

Y26C: *H. tarangensis*, *H. radiata* sp. nov., *H. vittaelatae* sp. nov., *H. micronesica* sp. nov.

GU44BF-1A: *H. dagmannii*, *H. coacervata* sp. nov., *H. schefteropsis* sp. nov.

GU52K-7: *H. alcyone*, *H. schefterae*, *H. carahii*, *H. coacervata* sp. nov., *H. schefteropsis* sp. nov.

GU52X-5: *H. spathulatoides*, *H. asteropeae*, *H. sinuosa* sp. nov.

GU66F-8: *H. contraria *sp. nov.

GU68-1B: *H. contraria* sp. nov., *H. dagmannii*

PN2-9: *H. corrugata*, sp. nov.

M2-13: ***H. dagmannii***, ***H. coacervata* sp. nov.**, *H. marshallensis* sp. nov., *H. majurana* sp. nov., *H. taygeteae*, *H. cf. volvendirostrata*
