# Supplementary figures and images for: The Search for Species Flocks in Marine Benthic Homoeocladia spp. (Diatomeae: Bacillariales). I. Variations on Three Themes, Seventeen New Species
Source: Plants (Basel). 2023 Dec 4;12(23):4073. doi: 10.3390/plants12234073 (PMC10708510; doi:10.3390/plants12234073)

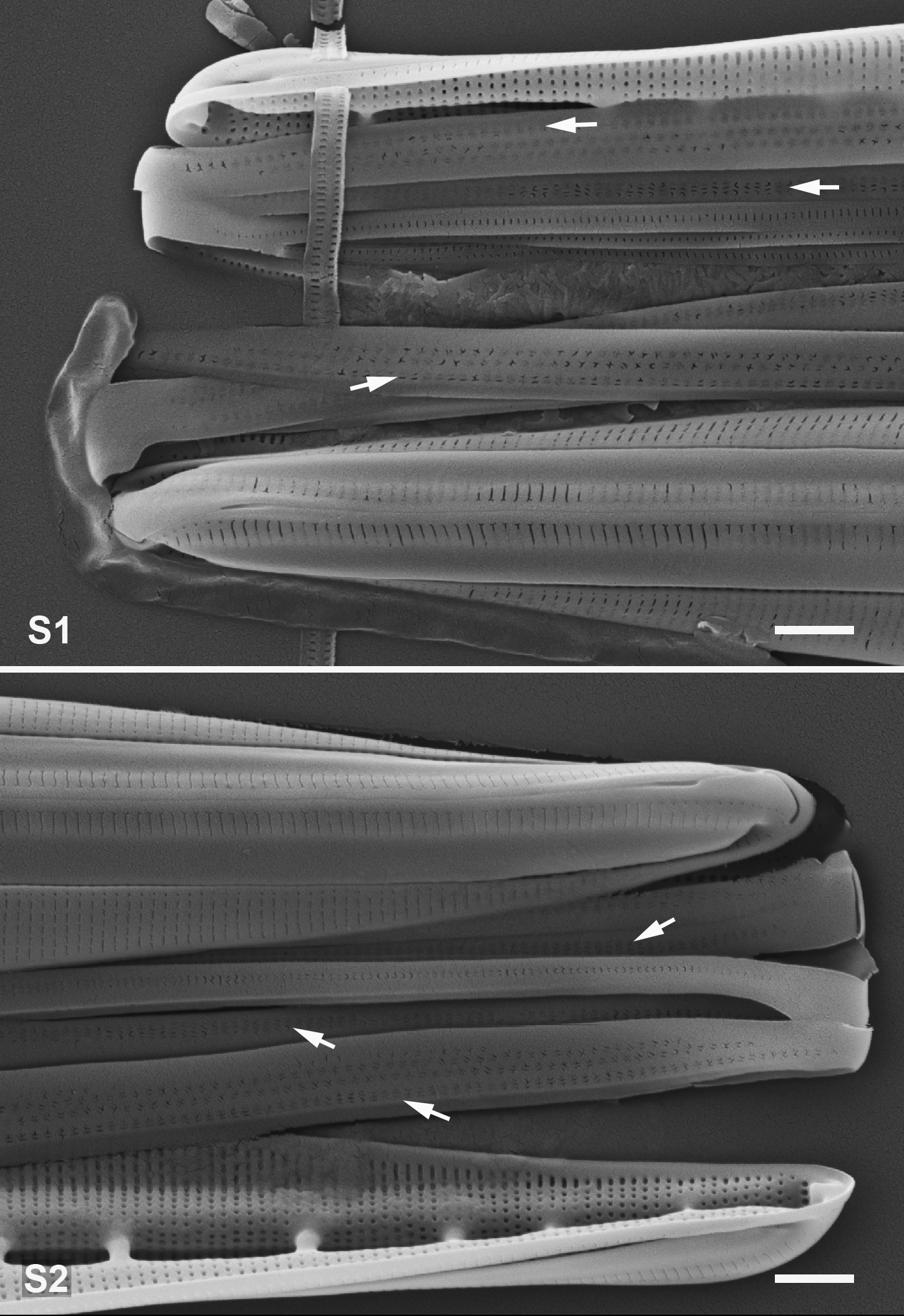

Supplement: Supplementary file 1 [file plants-12-04073-s001.zip › plants-2576879-supplementary.jpg]
